# Exercise Protects Skeletal Muscle Fibers from Age-Related Dysfunctional Remodeling of Mitochondrial Network and Sarcotubular System

**DOI:** 10.3390/cells15030248

**Published:** 2026-01-27

**Authors:** Feliciano Protasi, Matteo Serano, Alice Brasile, Laura Pietrangelo

**Affiliations:** 1 CAST, Center for Advanced Studies and Technology & DMSI, Department of Medicine and Aging Sciences, University Gabriele d’Annunzio of Chieti-Pescara, I-66100 Chieti, Italy; feliciano.protasi@unich.it (F.P.); alice.brasile@unich.it (A.B.); 2 Department of Molecular and Developmental Medicine, Molecular Medicine Section, University of Siena, I-53100 Siena, Italy; matteo.serano@unisi.it

**Keywords:** triad, Ca^2+^ release unit (CRU), excitation-contraction (EC) coupling, mitochondria, sarcoplasmic-reticulum (SR), store-operated Ca^2+^ entry (SOCE), transverse tubule (TT)

## Abstract

**Highlights:**

**What are the main findings?**

- Studying the effect of inactivity vs. exercise on ultrastructure of skeletal muscle fibers has given us the opportunity to discover that the correct position of mitochondria is lost in sedentary aging and inactivity, but is maintained by regular exercise.
- Function of SOCE (a mechanism that allows fibers to use external Ca^2+^ and limit muscle fatigue) also depends on regular muscle activity.

**What are the implications of the main findings?**

- The maintenance of the internal architecture of muscle fibers is crucial for their capability to function properly.
- The proper position of mitochondria in proximity of sites of Ca^2+^ release and Ca^2+^ entry may be crucial for the metabolic efficiency of muscle, and thus of the entire organism.

**Abstract:**

In skeletal muscles fibers, cellular respiration, excitation–contraction (EC) coupling (the mechanism that translates action potentials in Ca^2+^ release), and store-operated Ca^2+^ entry (SOCE, a mechanism that allows recovery of external Ca^2+^ during fatigue) take place in organelles specifically dedicated to each function: (a) aerobic ATP production in *mitochondria*; (b) EC coupling in intracellular junctions formed by association between transverse tubules (TTs) and sarcoplasmic reticulum (SR) named *triads*; (c) SOCE in *Ca*^2+^ *entry units* (CEUs), SR-TT junctions that are in continuity with membranes of triads, but that contain a different molecular machinery (see Graphical Abstract). In the past 20 years, we have studied skeletal muscle fibers by collecting biopsies from humans and isolating muscles from animal models (mouse, rat, rabbit) under different conditions of muscle inactivity (sedentary aging, denervation, immobilization by casting) and after exercise, either after voluntary training in humans (running, biking, etc.) or in mice kept in wheel cages or after running protocols on a treadmill. In all these studies, we have assessed the ultrastructure of the mitochondrial network and of the sarcotubular system (i.e., SR plus TTs) by electron microscopy (EM) and then collected functional data correlating (i) the changes occurring with aging and inactivity with a loss-of-function, and (ii) the structural improvement/rescue after exercise with a gain-of-function. The picture that emerged from this long journey points to the importance of the internal architecture of muscle fibers for their capability to function properly. Indeed, we discovered how the intracellular organization of the mitochondrial network and of the membrane systems involved in controlling intracellular calcium concentration (i[Ca^2+^]) is finely controlled and remodeled by inactivity and exercise. In this manuscript, we give an integrated picture of changes caused by inactivity and exercise and how they may affect muscle function.

## 1. Introduction

The main function of skeletal muscle in the human body is to generate force and movements of the skeleton. Though, skeletal muscle also has other important roles such as maintaining thermoregulation and controlling metabolic balance of individuals. Why skeletal muscle is so important for the metabolic balance is because muscle fibers contain the majority of mitochondria of the entire body. Mitochondria are the intracellular organelles dedicated to cellular respiration and their efficiency in consuming oxygen and thus producing ATP aerobically is crucial for the ability of our organism to burn the diet caloric intake. For skeletal muscle to be efficient in using oxygen, its relative percentage of body mass must be in the normal range (between 50 and 60% of the body weight), muscle fibers must contain enough mitochondria, and mitochondria must function properly. In turn, mitochondrial function is controlled by intracellular calcium concentration (i[Ca^2+^]), the divalent cation that also activates muscle contraction. The fact that an active lifestyle is the ideal approach to maintain a healthy skeletal muscle system is widely accepted. It is also agreed that maintenance of muscle mass requires resistance training (i.e., exercise against a load that provides resistance to the movement), while proper mitochondrial function is mainly controlled by aerobic exercise (i.e., exercise against low resistance for extended periods of time). Our experience in the past 20 years in (i) studying the ultrastructure of skeletal muscle fibers during postnatal maturation, adulthood, and aging and (ii) assessing the effect of inactivity vs. exercise has given us the opportunity to understand more about a new aspect of muscle adaptation to exercise that has been overlooked: the importance of mitochondrial intracellular position and of their interaction with the sarcotubular system, i.e., the membrane systems controlling i[Ca^2+^].

## 2. Organization of Organelles and Intracellular Membranes Involved in Aerobic ATP Production and Ca^2+^ Handling

Calcium ions (Ca^2+^) are extremely versatile intracellular messengers that control many physiological functions including mitochondrial activity [1,2,3,4,5,6]. Entry of Ca^2+^ in the mitochondrial matrix (defined as *excitation–metabolism coupling*) stimulates the respiratory chain [7,8,9,10] and relies on a bi-directional communication between the sarcoplasmic-reticulum (SR) and mitochondria that involves Ca^2+^ ions and reactive oxygen species (ROS) signaling [7,8,10,11,12,13,14].

Calcium handling in muscle fibers depends on several mechanisms, the most important being (a) release of Ca^2+^ from SR in response to action potentials delivered by motor neurons, which controls the contraction and relaxation of the contractile elements—a mechanism known as *excitation–contraction (EC) coupling* [15,16,17,18,19,20,21]; and (b) entry of Ca^2+^ from the extracellular space following repetitive stimulation that causes SR depletion, defined as *store-operated Ca*^2+^ *entry (SOCE)* [22,23,24,25,26,27,28,29,30,31,32].

Each of the different mechanisms described above uses different intracellular membrane systems and organelles: (a) mitochondrial uptake of Ca^2+^ during excitation–metabolism coupling likely relies on the position of mitochondria next to sites of SR release [11,12,33,34,35,36,37,38], and possibly also to sites of SOCE (see below); (b) EC coupling is mediated by *triads*, also known as *Ca*^2+^ *release units* (CRUs), specialized intracellular junctions formed by transverse tubules (TTs, invaginations of the surface membrane that carry the action potential into the fiber interior) and specialized domains of the SR (i.e., the terminal cisternae) dedicated to accumulate and release the Ca^2+^ needed to activate contraction [39,40,41,42,43]; finally (c) SOCE occurs in *Ca*^2+^ *entry units* (CEUs), also junctions between SR and TT, that are few and small in rest condition, but that grow in number and size during exercise [44,45,46,47,48,49]. Mitochondria, triads, and CEUs in adult/healthy fibers retain a specific intracellular position, which is dictated by the dark–pale cross-striation, which in turn is generated by the lateral alignment of contractile elements, i.e., the *myofibrils*.

### 2.1. Organelles Dedicated to Aerobic ATP Production: Mitochondria

Adenosine triphosphate (ATP) is the source of energy for many cellular functions. In muscle fibers, ATP (i) allows the dissociation of the myosin heads from actin; (ii) provides the energy needed for the power stroke, hence force generation; (iii) allows the relaxation of fibers thanks to Ca^2+^ reuptake operated by SERCA pumps (sarco-endoplasmic reticulum Ca^2+^ ATPases) [50,51]; (iv) re-establishes the proper balance of Na^+^ and K^+^ following the propagation of action potentials thanks to Na^+^/K^+^ ATPases in the plasma membrane [52]. As ATP is continuously consumed, the cells must be capable of producing new ATP in a fast and efficient manner. Consumed ATP can be replenished either anaerobically or aerobically: the anaerobic alactic (meaning that it does not produce lactic acid) and lactic systems replenish ATP without the need of oxygen [53], while the aerobic system replenishes ATP in the presence of oxygen. The aerobic metabolism occurs within the mitochondrial matrix and represents the major source of cellular ATP production in muscle providing about 90% of all cellular metabolism [54]. Aerobic metabolism is the process by which carbohydrates and fatty acids are converted into energy in the presence of oxygen [55].

It has been proposed that mitochondria have a bi-directional interaction with the SR in adult skeletal muscle fibers, an orthograde and a retrograde communication: (a) SR-to-mitochondrion signaling enhances aerobic ATP production through Ca^2+^ influx into the mitochondrial matrix [56,57,58]; whereas (b) mitochondrion-to-SR signaling supposedly uses ROS to suppress SR Ca^2+^ release [59,60]. This crosstalk has also been defined as excitation–metabolism coupling [12,34].

As ATP production during mitochondrial respiration uses mechanisms stimulated by elevations in [Ca^2+^] in the mitochondrial matrix [5,57,61], the activation of muscle contraction (induced by transient elevation in i[Ca^2+^]) also serves as a trigger to boost aerobic ATP production needed to keep pace with the increased crossbridge cycle activity and SERCA-mediated Ca^2+^ removal from the cytoplasm. While it was reported that contractile relaxation was slower in more glycolytic fibers (poor in mitochondria) than in slow-twitch fibers (rich in mitochondria) [62], suggesting that under physiological conditions mitochondria may influence the temporal development of Ca^2+^ transients, the fact that mitochondria accumulate large amounts of Ca^2+^ (suggested by some authors) remains controversial. This is because, on the one hand, the [Ca^2+^] required for mitochondrial Ca^2+^ transport is considerably higher than the one achieved during Ca^2+^ transients elicited by EC coupling (only ~1–2 µM), while, on the other hand, the relative percentage of fiber volume occupied by mitochondria is relatively small, ranging from about 3–4% to approximately double, respectively, in fast- vs. slow-twitch fibers [33,63,64]. Abnormalities in Ca^2+^ handling and inefficient aerobic ATP production by mitochondria underline several physio-pathological conditions, such as dysfunction in myopathies, compromised metabolic balance, skeletal muscle fatigue, and reduced endurance in aging. While different mechanisms were hypothesized for entry of Ca^2+^ into the mitochondria [65,66,67], the identification of a mitochondrial Ca^2+^ uniporter (MCU) may have revealed the main pathways for mitochondrial Ca^2+^ uptake [68,69,70]. While the [Ca^2+^] needed for Ca^2+^ uptake in isolated mitochondria is about 10 μM [71], studies that directly measured mitochondrial Ca^2+^ in intact skeletal fibers [7,11] proposed that this uptake occurs also during single twitches and tetanic stimulation [8], conditions in which [Ca^2+^] does not rise as high. Physiologists have tried to reconcile this apparent discrepancy, postulating the concept of *local Ca*^2+^ *microdomains*, i.e., narrow spaces inside muscle fibers in which [Ca^2+^] may rise high enough in proximity of mitochondria before Ca^2+^ will diffuse away to myofibrils. For this to happen, mitochondria would have to be strategically positioned in proximity to the sites of Ca^2+^ release. Indeed, an intimate structural interaction between mitochondria and endoplasmic/sarcoplasmic reticulum (ER/SR) has been identified in non-muscle cells [72,73], in cardiomyocytes [74], and finally also in skeletal muscle fibers [33].

**Figure 1:**
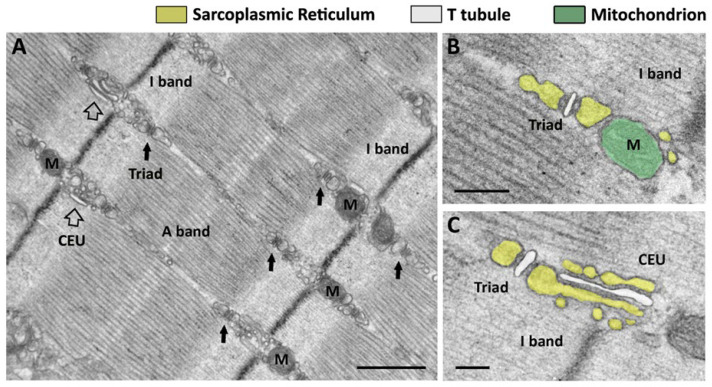
Disposition (panel (**A**)) and morphology (panels (**B**,**C**)) of mitochondria, triads, and Ca^2+^ entry units in adult skeletal muscle fibers. (**A**) Skeletal muscle fibers are striated cells (i.e., characterized by alternating I and A bands) in which mitochondria (M), triads (small arrows), and Ca^2+^ entry units (CEUs, empty arrows) occupy a specific position: mitochondria and CEUs at the I band, while triads are placed in proximity of the transition between I and A bands. (**B**,**C**) At higher EM magnification, we have a false-colored triad (formed by a central TT flanked by two SR terminal cisternae) and a closely associated mitochondrion (panel (**B**)) and a representative CEU, the site of SOCE in skeletal muscle, next to a triad (panel (**C**)). Scale bars: (**A**) = 0.5 μm; (**B**,**C**) = 0.1 μm.

In mammalian skeletal fibers, most mitochondria (M in Figure 1A)seem to occupy a preferential position in proximity to Z lines, in correspondence of the pale band (named I band from *isotropic*) generated by the lateral alignment of myofibrils. Already in 1985, Ogata and Yamasaki, in an elegant study performed using EM, described a population of intermyofibrillar mitochondria surrounding myofibrils as incomplete rings on both sides of the Z line [75]. These I band mitochondria were present in all types of fibers, i.e., fast, intermediate, and slow in adult rat leg muscles. It should be acknowledged that in slow-twitch fibers, there are also other populations of mitochondria: some under the sarcolemma in proximity of capillaries and others forming longitudinal columns between myofibrils next to the A band [75]. In 2009, we described how I band mitochondria in skeletal muscle fibers are placed in proximity to sites of Ca^2+^ release, the triads [11,33], and detected the presence of small electron-dense strands (generically named *tethers*) between the outer mitochondrial membrane and the SR. Tethers must provide a strong link between the two organelles, as treatment with hypotonic solution causing swelling of the cytoplasmic space was unable to disrupt the SR–mitochondria association [33]. The molecular identity of tethers remains to be determined, even if a possible contribution of Mitofusin-2 was proposed [76]. Interestingly, before reaching their final position at the I band in adult fibers, the majority of mitochondria in the first weeks after birth in mice are disposed longitudinally between myofibrils. The shift in the mitochondrial network from longitudinal to transversal during post-natal maturation of skeletal fibers mimics (but with a temporal delay) the maturation of the EC coupling apparatus, in which TTs are initially longitudinal before becoming completely transversal (see Section 2.2 for additional detail).

### 2.2. The Organelles Dedicated to Ca^2+^ Handling: Triads and Ca^2+^ Entry Units

*a. The sites of Excitation-Contraction (EC) Coupling: Triads (also named Ca*^2+^ *Release Units, CRUs).* EC coupling is a physiological event that converts the propagation of an action potential in the sarcolemma into a mechanical response of muscle fibers (named single twitch) due to the release of Ca^2+^ from the SR, which activates contractile elements [19,77,78,79,80], i.e., the myofibrils. Myofibrils are constituted by repeating contractile units, the sarcomeres, in which thick and thin filaments generate alternating dark A bands and pale I bands [9,81,82]. The fact that the contraction and relaxation of muscle fibers was the result of the release and reuptake of Ca^2+^ ions from the SR has been known long before the molecular machinery controlling EC coupling was discovered [83,84,85,86,87]. In skeletal muscle fibers, EC coupling is defined as mechanical, describing a direct communication between TTs that carry the electrical signal and the SR containing Ca^2+^ [77,88,89,90,91]. *Mechanical EC coupling* is the evolution of *Ca*^2+^*-induced Ca*^2+^ *release (CICR)*, a more primitive EC coupling mechanism utilized by cardiac and smooth muscle cells, where the entry of Ca^2+^ from the extracellular space activates Ca^2+^ release from SR stores [92,93,94,95,96,97,98,99]. Mechanical EC coupling relies on the direct communication between two proteins: (a) skeletal dihydropyridine receptors (DHPRs), voltage-gated L-type Ca^2+^ channels of external membranes that in skeletal muscle mainly acts as voltage sensors [88,89,100,101,102,103,104], and (b) ryanodine receptors type-1 (RYR1), the main isoform of SR Ca^2+^ release channel expressed in adult skeletal muscle fibers [18,105,106,107,108,109,110]. The direct association between one RYR1 in the SR terminal cisternae (formed by four identical subunits) and four DHPRs in the TT membrane was demonstrated by the discovery of tetrads, intramembrane particles visualized in freeze-fracture replicas of TTs [78,110,111,112,113,114,115,116,117]. The fact that a tetrad is formed by four DHPRs mechanically coupled to one RYR1 was proved in experiments showing how (i) tetrads are missing in dysgenic muscle (a natural mutation causing ablation of the alpha1 subunit of the DHPR) [42]; and (ii) DHPRs do form tetrads in dyspedic muscle (a genetically engineered knockout of RYR1), but restored by transfection of dyspedic cells with RYR1 [113]. In mechanical coupling RYR1, Ca^2+^ release channels are directly opened by four voltage sensors contained in a DHPR-tetrad. While DHPR-tetrads and RYR1 are unquestionably the two main players in the skeletal EC coupling, many other proteins modulate and coordinate their interaction. Among them, calsequestrin, triadin, junctin, junctophilins, FKBP12, and STAC3 are the proteins more studied in the field [118,119,120,121,122,123,124,125,126,127,128]. All those proteins, together with RYR1 and DHPR, constitute a macromolecular complex that regulates the activation of Ca^2+^ release from the SR terminal cisternae, i.e., EC coupling.

The molecular assembly of all those proteins requires the association of membranes carrying the action potential to those that store Ca^2+^: this association occurs in specialized intracellular junctions, known as Ca^2+^ release units (CRUs), formed by the association of (a) invaginations of external membranes known as TTs [129], which carry the depolarization of the sarcolemma (triggered by the motor neuron at the neuromuscular junction, NMJ) and contain DHPRs; and (b) terminal cisternae, specialized SR domains that are decorated by feet, the cytoplasmic domain of RYRs, and contain calsequestrin (CASQ). CASQ is the main SR buffer that allows terminal cisternae to accumulate the large amounts of Ca^2+^ needed to generate tetanic contractions, required for skeletal muscle function [130]. CRUs in adult skeletal muscle fibers are named triads because they are formed by three elements: a central TT—showing a narrow and flat profile—associated with two lateral terminal cisternae (Figure 1B,C) [39]. Triads are the final result of differentiation and maturation that occurs while myotubes become adult fibers: (a) at first, CRUs are formed by only two elements, in which the interaction DHPR-RYR occurs in peripheral couplings formed between the SR and the sarcolemma; (b) then, once the sarcolemma invaginates to form a primitive TT network, the junctional SR associates with TTs that are initially longitudinally oriented, i.e., parallel to the long axis of myofibrils [40,41,42]; finally, (c) when the TT network becomes transversal and forms two networks that are positioned approximately at the transition between A and I bands of relaxed sarcomeres (approximately 2.2–2.4 µm long), CRUs become transversal triads. This position of triads is common to all mammalian muscles and is retained both in fast- and slow-twitch fibers [113] (small arrows in Figure 1A).

Looking at triads at higher magnifications by electron microscopy (EM), two structural details are visible: (a) the presence of dark electron densities in the junctional gap between TT and SR, representing feet, the cytoplasmic domain of RYRs. RYR-feet in triads of mammals usually form two rows in which individuals RYRs touch each other corner-to-corner [131] (feet are visible in Figure 1B as densities between TT and SR, respectively, labeled in white and yellow); and (b) the lumen of SR terminal cisternae filled with dark matrix that reveals the presence of CASQ, the main SR Ca^2+^ buffer accumulating Ca^2+^ in proximity to the sites of release [118,119,122,132]. C. Franzini-Armstrong, using deep-etch replicas, demonstrated that the CASQ matrix is anchored at the SR terminal cisterna via thin, long strands, supposedly triadin or junctin molecules [133,134].

Contrary to RYR-feet, DHPR voltage sensors could not be visualized in standard EM because they are almost entirely embedded within the TT lipid bilayer. C. Franzini-Armstrong used a different technique, freeze-fracture replicas, to discover that in skeletal muscle DHPRs were grouped in four particles [79,111,131] and demonstrated that DHPRs need association with RYR1 subunits to form tetrads [112,113]. This assembly is peculiar to skeletal muscle cells because, in smooth and cardiac muscle, which express different RYR isoforms (RYR2 and RYR3), tetrads are missing. The presence or absence of DHPR-tetrads has been related to EC coupling being either mechanical or CICR. Indeed, re-expression of RYR1, but not RYR2 or RYR3, in dyspedic myotubes (i.e., RYR1-knockout) restored tetrads arrangements of DHPRs, which otherwise were randomly disposed [114,115].

*b. The sites of Store-Operated Ca*^2+^ *Entry (SOCE): the Ca*^2+^ *Entry Units (CEUs)*. The importance of external Ca^2+^ for the proper function of skeletal muscle fibers has been overlooked for many years because of EC coupling being mechanical (see above), i.e., it does not rely on CICR as cardiac or smooth muscle. Nonetheless, two forms of Ca^2+^ entry have been characterized in skeletal fibers:

- *Excitation-coupled Ca*^2+^ *entry* (ECCE), a pathway first described in the early 2000s, which was later associated with the opening of the alpha-1 subunit of the DHPR—the voltage sensor in mechanical EC coupling that also forms an L-type Ca^2+^ channel that (in skeletal fibers) is slow and has little ion conductance [135,136]. As ECCE is altered in malignant hyperthermia susceptibility (MHS), it was argued that it may contribute to the dysfunctional Ca^2+^ signaling found in muscle fibers of MHS patients [137].
- *Store-operated Ca*^2+^ *entry* (SOCE), a pathway that allows extracellular Ca^2+^ to enter the cytosol and refill SR stores during repetitive muscle activity [22,23,27,28,138]. SOCE is activated by a phenomenon known as *SR depletion*, i.e., a reduction in the total amount of Ca^2+^ stored in the SR caused by the loss of intracellular Ca^2+^ across the sarcolemma during prolonged muscle activity [139,140,141,142]. SOCE was first characterized in non-excitable cells [22,23], but was detected in skeletal muscle myotubes and fibers only several years later [24,25,26]. Initially, the molecular players of SOCE remained elusive for years after the first identification of the mechanism, until, in 2005–2007, the two main proteins involved were discovered in patients affected by a severe immunodeficiency [143,144,145,146,147]: (a) STIM1 (stromal-interacting molecule-1), an ER/SR protein that acts as Ca^2+^ sensors due to the presence of an intraluminal N-terminal EF-hand domain; (b) ORAI1, a Ca^2+^ release-activated channel (CRAC) of the plasma membrane. Also, calsequestrin-1 (CASQ1), a protein involved in EC coupling with the dual role of being the main SR buffer that accumulates Ca^2+^ in proximity to the sites of release and also a direct modulator of RYR1 [118,119,132], has been proposed to modulate SOCE in skeletal muscle [148,149,150].

In non-muscle cells, the proposed mechanism for activation of SOCE involves the depletion of intracellular stores (i.e., ER), which would induce Ca^2+^ dissociation from the luminal N-terminal EF-hand domain of STIM1 followed by conformational changes, dimerization, and relocation of STIM1 to sites of contact between ER and the plasmalemma. The association of ER containing STIM1 with the surface membrane (junctions defined as *puncta*) would allow aggregated STIM1 dimers to recruit, trap, and open ORAI1 and permit entry of Ca^2+^ from the extracellular space and replenishment of intracellular ER stores [143,144,145,146,147,151,152,153,154,155,156].

The importance of SOCE for skeletal muscle function and adaptation to exercise (i.e., contribute the Ca^2+^ needed to modulate muscle-specific gene expression and sustain internal SR Ca^2+^ stores, hence preventing muscle fatigue/weakness) started to become evident when the two main players in SOCE were also identified in striated skeletal fibers [157,158]. Lyfenko and Dirksen demonstrated that SOCE function in skeletal muscle fibers (but not ECCE) was dependent on STIM1 and ORAI1. Besides being important to counteract fatigue, it seems that SOCE may be important also for differentiation and development [159,160,161,162].

In skeletal muscle, during prolonged repetitive muscle activity, a small fraction of Ca^2+^ ions cycled by the SR during EC coupling is lost across the sarcolemma due to either simple leak or active extrusion by Na^+^/Ca^2+^ exchangers or plasma membrane Ca^2+^ ATPases (PMCA) [139,140,141,142]. As a reduction in the amount of Ca^2+^ stored in the SR, i.e., SR depletion, is one of the factors that contributes to the onset of premature fatigue, muscle (which often needs to work for extended periods of time) has changed during evolution to adopt a system that helps SR function, i.e., SOCE, the pathway that allows extracellular Ca^2+^ to enter the cytosol and refill SR stores during muscle fatigue [27,31]. SOCE in skeletal muscles developed peculiar features compared to other tissues. First, its activation following SR depletion seems to be much faster. Indeed, while in non-muscle cell activation of ORAI1 channels needs tens of seconds [155], experiments in skeletal fibers suggested that Ca^2+^ influx can be activated very quickly (<1 s) [24,163,164]. To explain this rapid SOCE activation, the presence of pre-formed SR-TT junctions was hypothesized, in which STIM1 and ORAI1 could already be clustered before depletion [27]. A STIM1 splice variant highly expressed in skeletal muscle, STIM1-long, was proposed to mediate rapid SOCE activation, referred to as *phasic-SOCE* [165,166].

For more than a decade after the detection of SOCE in skeletal muscle fibers, it was speculated that SOCE would occur at the triad junction (i.e., the same sites of EC coupling; see above). The reasons underlying this speculation was the fact that in triads SR (presumably containing STIM1) and TT (invagination of the surface membrane that should contain ORAI1) are already preassembled, providing a convenient and suitable site for the fast-mode activation of skeletal SOCE [27]. Although this hypothesis was logical, this assumption ignored the fact that there was no direct evidence for the presence of STIM1 and ORAI1 in triads. Furthermore, the junctional space in triads already contains many proteins (junctophilins, FKBP12, triadin, junctin, mitsugumin, STAC3, etc.; see Section 2.2), which are tightly assembled around regular arrays of RYR-feet in the SR mechanically coupled to DHPR-tetrads in TTs. This macromolecular complex could in principle interfere with the migrations of STIM1 oligomers in the SR membrane to form puncta, which are needed to recruit ORAI1 channels in TTs.

To determine the most likely sites for STIM1-ORAI1 interaction during SOCE in adult skeletal muscle fibers, we employed EM combined with immunogold, immunofluorescence, and functional fatigue protocols [44]. The experiments were performed in adult mice (4 months of age) before (control group) and after the animals were subjected to a fatigue running protocol on a treadmill (exercised group). The idea underlying our experiments was to induce fatigue and SR depletion, stimuli that would trigger SOCE activation. Unexpectedly, immunofluorescence and immunogold labeling revealed that colocalization of STIM1 with ORAI1 was minimal in control conditions, with most of STIM1 placed in the SR at the I band, while ORAI1 was retained in TTs at the triad junctions, colocalized with EC coupling protein (i.e., RYR1) [44]. The fact that, under control conditions, STIM1 had negligible triadic localization was already evident, but not fully acknowledged, in a previous publication [31]. Parallel functional experiments showed that in muscles from control mice, the contribution of external Ca^2+^ to contractility was indeed limited. EM then provided evidence that explained how STIM-ORAI1 localization could increase during exercise: we discovered how a single bout of incremental exercise on a treadmill triggered a significant remodeling of both SR and TT at the I band, involving formation of SR stacks and elongation of TTs from triads into the I band (Figure 1C). This remodeling allowed the formation of new SR-TT junctions structurally and molecularly different from triads. These new SR-TT junctions did not contain RYR1, but colocalized STIM1 and ORAI1, promoted by the elongation of TT bearing ORAI1 into the I band (shown by a specific staining obtained with ferrocyanide [44]), where SR stacks contained aggregated STIM1. We proposed that these new SR-TT junctions would function as *Ca*^2+^ *entry units* (CEUs) during SOCE, because functional experiments showed that their presence in muscles isolated from pre-exercised mice resulted in increased resistance to fatigue in the presence of external Ca^2+^. This gain-of-function promoted by exercise-induced assembly of CEUs could be blocked by removal of external Ca^2+^ or by using SOCE inhibitors [167,168].

The final evidence that the new SR-TT junctions that assemble at the I band during exercise were indeed the sites of SOCE came from the measurements of the rate of Mn^2+^ quenching in isolated single FDB fibers—the gold standard technique used to measure the entry of divalent cations from the extracellular space following SR depletion. In these experiments, the rate of Mn^2+^ quench was significantly increased in FDB fibers isolated from mice after they were subjected to the fatigue protocol (i.e., with CEUs formed), while Mn^2+^ quench was abolished in fibers from ORAI1-knockout mice. In the same study, we also collected evidence that CEUs are dynamic junctions that promptly assemble during exercise but dissemble during the following hours of recovery [46]. The parallel between (a) structural evidence showing elongation of TTs toward SR stacks during exercise followed by retraction during recovery and (b) Mn^2+^ quench being either up or down regulated by these changes allowed to conclude that the formation of functional CEUs is dependent on the elongation of TTs and on the formation of contacts with SR stacks at the I band, hence making TT remodeling the key event in the process of formation of functional CEUs [46].

The mechanisms leading to the exercise-induced remodeling of SR and TT are still quite obscure. In Girolami et al. 2023, we demonstrated that (a) CEUs can assemble in isolated muscles, in complete absence of innervation and blood supply; (b) CEU assembly is pH- and temperature-sensitive: assembly is boosted by higher temperature and lower pH, two parameters that do change accordingly during exercise [169]. The fact that STIM1 has been proposed to function as a multipurpose stress transducer sensitive to changes like oxidation, temperature, hypoxia, etc. [170,171], may explain how changes in pH and temperature may boost CEU assembly during repetitive electrical stimulation ex vivo [169].

Why CEUs were never identified and characterized before is a puzzling question with an easy explanation. CEUs are few and quite small in muscles isolated from control mice (empty arrows in Figure 1A), and hence never identified as an independent functional units. Accordingly, muscle from control mice (not exercised) can use only little external Ca^2+^ during repetitive stimulation. In addition, immunolabeling for confocal or immunogold for EM to detect colocalization between STIM1 and ORAI1 was never performed with the level of resolution performed by Boncompagni and colleagues in 2017 and 2018 [44,50]. Additionally, the identification of CEUs was allowed by an experiment that was not performed before: fixation for EM and immunolabeling of EDL muscles from mice after they were subjected to an in vivo fatigue protocol designed to induce fatigue, and possibly SR depletion. Because the in vivo fatigue protocol on treadmill induced a striking increase in size and number of SR stacks, identification of the new structures in EM came from the simple comparison between muscles isolated from exercised mice and those from control mice, which only had few and small SR stacks. Complementary experiments then allowed the verification that these stacks also (i) contained elongated TTs, (ii) allowed increased colocalization of STIM1 and ORAI1, and (iii) boosted Ca^2+^ entry [172]. A commentary published in *J. Gen. Physiol.* proposed CEUs as *the backdoor for Ca*^2+^ *ions in muscle cells*, bringing new attention to the importance of external Ca^2+^ in the function skeletal fibers [173].

The discovery of CEUs between 2017 and 2019 also led to other significant findings and hypotheses:

(a) Since we generated CASQ1-knockout mice about 20 years ago [174], we have been always puzzled by the fact that these mice could live quite normally without CASQ1, hence with reduced Ca^2+^ stored in the SR (not considering the fact they were oversensitive to heat [175,176]). In 2010, we reported that CASQ1-knockout fibers undergo SR depletion under high frequency stimulation [177]. Only in 2020 did we come to the final conclusion that CASQ1-knockout mice could live a quite normal life because, during post-natal development, skeletal fibers progressively adapted by downregulating CASQ2 expression (that is not accompanied by increased expression of CASQ1, because this gene in knocked out [178]) and assembling constitutive CEUs [45]. The constitutive presence of CEUs would allow muscle fibers lacking CASQ1 to deal with the insufficient storage of Ca^2+^ in the SR, constantly supplementing internal function with external Ca^2+^ coming into the fibers via the constitutively present CEUs.

(b) It has been suggested by different authors that SOCE is compromised in aging [179,180]. In 2021, we showed a lack of structural elements that allow assembly of CEUs in muscle of sedentary aged mice (i.e., SR stacks and elongated TTs at the I band) that could explain dysfunctional SOCE [181]. However, we also showed that CEUs could be rescued in mice exercising voluntarily in wheel cages, suggesting that regular exercise while aging could maintain SOCE functionality later in life.

(c) The presence of CEUs, and hence gain of SOCE function, may increase the risk of heat stroke in extreme climatic conditions. Indeed, their constitutive assembly in CASQ1-null mice resulted in an exertional-environmental heat stroke (EHS) phenotype, i.e., mice are susceptible to trigger lethal episodes in conditions of high temperature and/or strenuous exercise [29,44,176,177,182]. Also, exercise-induced assembly of CEUs in wild-type mice contributed to the increase in body temperature during exertional stress, predisposing them to a possible heat illness [169].

## 3. Sedentary Aging Compromises the Architecture of Mitochondrial Network and Sarcotubular System

Aging is a physiological process that causes structural and functional decay of many functions in the human body [183,184,185,186]. The age-related decline of neuromuscular function is likely the change that most affects the quality of life of elderlies, also causing a dramatic increase in the health care costs, because it impairs independence in daily activities [187,188,189,190,191,192,193]. The main effect of sedentary aging on skeletal muscle is *sarcopenia* (a phenomenon causing loss of 40–50% of muscle mass between the ages of 30 and 70), which represents the combined result of a variety of changes including loss of motor units due to progressive denervation of fast-twitch motor units, atrophy of remaining fibers, oxidative stress, etc. [194,195,196,197].

With increasing age, beside sarcopenia, muscle lose resistance to fatigue and force in all individuals, with different degrees of severity [195,198,199,200]. The explanation for the diminished resistance to fatigue is straight forward, as aging is accompanied by a great impairment of mitochondria at various levels: a reduction in mitochondrial volume and number is well documented in the literature, as well as the alteration of mitochondrial fusion and fission dynamics [201,202,203,204]. Regarding the reduced force output, the issue is slightly more complicated, as [202,205,206] the age-related decay of *specific force* (SF, i.e., the force produced by the muscle normalized to its cross-sectional area, CSA) is greater than the loss of muscle mass, i.e., atrophy. Delbono and colleagues proposed an impairment in EC coupling, named *EC uncoupling*, to explain a diminished supply of Ca^2+^ ions to myofibrils [207,208,209,210,211].

To probe the scientific issues presented above from a different perspective, we studied mitochondria and the EC coupling system in human biopsies from elderlies and in muscles isolated from aging mice [48,64,212,213,214]. Additionally, we also studied a further aspect that may contribute to loss of specific force in aging, proposing that dysfunctional SOCE could impair contractile function during repetitive stimulation [48,63,64,172,181,212,213].

### 3.1. Uncoupling of Mitochondria from Triads with Increasing Age

**Figure 2:**
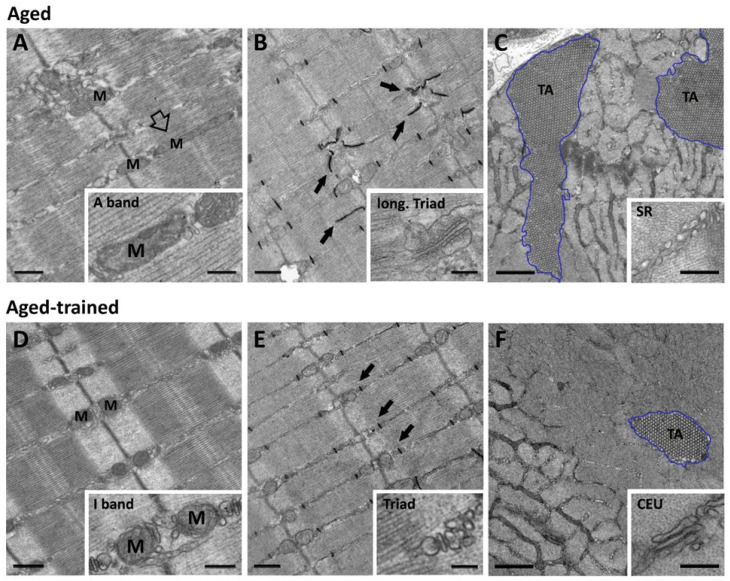
Longitudinal (panels (**A**,**B**,**D**,**E**)) and transverse (panels (**C**,**F**)) EM sections of skeletal muscle fibers from aged (panels (**A**–**C**)) and aged-trained (panels (**D**–**F**)) mice. (**A**–**C**) In fibers from aged mice, (a) some mitochondria (M) are misplaced at the A band of the sarcomere (empty arrow in panel A, and inset); (b) TT network (identified by a dark precipitate in panel (**B**)) and triads (inset) may become oblique or longitudinally oriented (small arrows in panel (**B**), and inset); (c) SR may form TAs (outlined in blue in panel (**C**)) and rarely form SR stacks at the I band (inset in panel (**B**)). (**D**–**F**) In fibers from aged-trained mice, (a) mitochondria (M) are correctly positioned at the I band (M in panel (**D**), and inset); (b) TT network (identified by a dark precipitate in panel (**C**)) and triads (inset) are transversally oriented (small arrows in panel (**E**) and inset); (c) TAs (outlined in blue in panel (**F**)) are diminished in number and size, while SR at I band more often forms SR stacks (inset in panel (**F**)). Scale bars: (**A**,**B**,**D**,**E**) = 0.5 μm; (**C**,**F**) = 1 µm, insets (**A**,**D**) = 0.25 μm, insets (**B**,**E**) = 0.1 μm, insets (**C**,**F**) = 0.3 μm.

In two studies performed with traditional bidimensional EM techniques—one performed in human biopsies (from individuals of 70 ± 4 years of age) and the other in muscles from aging mice (of 2 years of age)—we analyzed the number, volume, and position of mitochondria [63,64,212,214,215,216]. The results collected in the two different models were similar, suggesting that the findings could be transversal to different species: (a) the number and volume of mitochondria decrease significantly with age; (b) a fraction of mitochondria moves away from their normal position at I band (where they should be coupled by tethers to triads; Figure 1B) to become more longitudinally oriented at the A band (empty arrow in Figure 2A). Recently, elegant studies performed using electron and confocal microscopy also showed modification of three-dimensional architecture of the mitochondrial network in human fibers between young and older individuals [214,215,216]. In skeletal fibers of young individuals, the mitochondrial network appeared more complex than that of older individuals, where mitochondria structure shifts to a mostly compact, less complex, and spherical phenotype [216].

This misplacement of mitochondria from their proper I band position (together with the decrease in total number of mitochondria and triads; see Section 3.2 below) reported in our work [63,64,212] contributes to the reduced number of functional mitochondria–triad couples, reported both in human samples and in muscles from mice [63,212]. We described that mitochondria are connected to triads by tethers, little linkers that do not yet have a defined molecular identity [76]: the number of tethers was decreased in aging mice [63], a parameter that may underline the decrease in mitochondria–triad couples also detected in human biopsies [212]. In turn, the total number of mitochondria–triad couples could in principle affect the proper crosstalk between the two organelles, supposedly based on Ca^2+^ signaling from triad to mitochondria and ROS signaling from mitochondria to triads [11,12,33,34,35].

The mouse study also contained experiments performed with confocal microscopy, functional and biochemical measurements of mitochondrial Ca^2+^ uptake, and measurements of membrane potential and oxidative stress [63]. These experiments (a) correlated structural modifications with functional changes, i.e., reduced mitochondrial Ca^2+^ uptake; and (b) allowed us to hypothesize that elevated oxidative stress could contribute to the structural modifications of mitochondria (and of triads; see Section 3.2) by causing damage to membranes and proteins.

### 3.2. Reductions in Number of Ca^2+^ Release Sites, i.e., Triads

Muscle fiber contraction is triggered by rapid increases in i[Ca^2+^], known as transients. *Ca*^2+^ *transients* represent the summation of many Ca^2+^ release events, known as *Ca*^2+^ *sparks* [217,218,219]. It has been argued that each individual Ca^2+^ spark arises from a release site [60,220,221,222,223], which could be reasonably identified in a single triad (or CRU). As detailed in Section 2, triads in adult muscle are positioned next to the I-A transition of sarcomeres, when they are relaxed (small arrows in Figure 1A). In 2006, we analyzed, using transmission electron microscopy, the EC coupling system in human muscle biopsies of adult (average age 31 ± 3) [213] and aged individuals (average age 70 ± 4) in an attempt to find a possible structural explanation to the reduction in Ca^2+^ supply to contractile elements in aging muscle [207,208]. The results collected showed that sedentary aging results in a progressive disarray of membranes involved in EC coupling, which may have different degrees of severity in different fibers and/or in different areas of the same fiber [213]. The modifications to the Ca^2+^ handling system consist primarily of (a) progressive disarrangement of triads (sometimes formed by only two elements instead of three, i.e., dyads), which may be often improperly oriented (oblique or longitudinal instead of transversal) (arrows in Figure 2B); and (b) a significant reduction (about 30–40%) in the overall number of triads available for activating contraction of myofibrils. The loss of Ca^2+^ release sites in aged specimens could contribute to the inefficient delivery of Ca^2+^ ions to myofibrils, hence to EC uncoupling, as proposed by Delbono and colleagues [207,208]. A lower number of triads would lower the number of Ca^2+^ sparks in response to stimulation by the motor neuron, which, in turn, could result in inefficient generation of a Ca^2+^ transient and consequent impaired activation of the contractile machinery. Similar results were also collected in a second study analyzing human biopsies of sedentary individuals [212] and were reinforced by results in aging mice, animals of 2 years of age kept in normal cages (with no access to wheels for running): a reduction in the total number of triads and loss of proper orientation due to the increased presence of longitudinal TTs (arrows and inset in Figure 2B). The lower number of triads in Zampieri et al., 2015 [212] and Pietrangelo et al., 2015 [63] (i) were correlated with functional experiments showing impaired function in humans and reduced force output in mice and (ii) contributed to the reduced number of mitochondria–triad pairs (see above, Section 3.1).

### 3.3. Formation of Tubular Aggregates (TAs) and Loss of Ca^2+^ Entry Units (CEUs)

Tubular aggregates (TAs) are regular arrays of long SR tubes, which have been found in a variety of human disorders, including TA myopathy (TAM) [224,225,226,227,228,229]. TAM is a muscle disease that have been linked to mutations in STIM1, ORAI1 [230,231,232,233,234,235], and CASQ1 [236], the main players in SOCE. Interestingly, TAs have also been found in fast-twitch fibers of male aging mice [134,237] (outlined in blue in Figure 2C), but to our knowledge, their presence has not been confirmed in biopsies of healthy aging humans. Mechanisms underlying formation of TAs are still under investigation: several years ago, it was demonstrated that formation of TAs may be induced by anoxia [238], while in our experience, inactivity (which can also result in anoxia) is a contributing factor [181].

In two papers published, respectively, in 2012 and 2021, we showed that TAs in aging mice stain positive for STIM1, ORAI1, and CASQ1, but (as TTs and triads are excluded from TAs) negative for RYR staining [135,181]. Accumulation of STIM1, ORAI1, and CASQ1 in TAs of aging mice correlates with functional experiments showing an increased fatigability of muscles during repetitive stimulation and dysfunctional SOCE [181]. Inactive SOCE in aging muscle was assessed in isolated EDL muscles using experiments performed in the presence or absence of extracellular Ca^2+^, where aging muscles (i.e., that contain TAs) were unable to use extracellular Ca^2+^ via SOCE during fatigue protocols [181]. Structurally, the presence of TAs also correlated with the absence of SR stacks and elongated TT at the I band (inset in Figure 2C), the two structural components of junctions (i.e., CEUs) dedicated to SOCE (see above Section 2.2).

The conclusion that emerged from this study [181] was that TAs could represent a dysfunctional remodeling of the SR that traps (also) SOCE proteins in a dysfunctional compartment. This SR remodeling could be the consequence of aging with reduced muscle activity (i.e., mice were kept in standard cages, with no access to wheels for voluntary training), which means mice were aging with reduced opportunity to keep active, fatigue muscle, and recruit SOCE (which possibly could be inactivated because it is not necessary for everyday life). These findings are in line with previous publications by other investigators showing how a reduction in SOCE activity contributes to age-related muscle weakness [179,180,239].

## 4. Regular Exercise Prevents Age-Dependent Damage to Mitochondria and Membrane Systems Involved in Ca^2+^ Handling

It is generally agreed that skeletal muscle has great plasticity and can promptly respond to different stimuli, the most potent being exercise. While the effect of exercise on muscle mass and aerobic capacity has been widely studied [240,241,242,243,244], the new challenge is to unravel the intracellular signaling that leads to muscle adaptation to exercise, and to determine the different signaling pathways that mediate the responses to different types of exercise [245,246,247,248]. There is general consensus about the fact that adaptation of muscle to exercise will be different depending on which stimulus is challenging the muscle. To simplify this issue, exercise may be divided into two main categories: *resistance* vs. *endurance training*. Resistance training (i.e., exercise against a load that provides resistance to the movement, e.g., weight lifting, body building, sprint running, etc.) recruits preferentially fast-twitch fibers (type II) and is known to induce muscle hypertrophy; endurance training (i.e., exercise against low resistance for extended periods of time, e.g., walking, running, biking, cross-country skiing, etc.) is better suited to recruit slow-twitch fibers (type I) and will promote changes that increase the aerobic capacity of muscle (increase in mitochondrial number/volume, myoglobin expression, vascularization, etc.) [249,250,251,252,253]. Considering our interest in the remodeling of the mitochondrial network in response to different stimuli, we studied the effect of long-term endurance training on the mitochondrial network and on their association to triads in biopsies from elderly sportsmen and in aged mice. To do so, we collected samples as follows: (a) muscle biopsies from well-trained seniors (average of 70 ± 4 years of age); (b) isolated EDL muscles from 2-year-old mice that trained voluntarily for 1 year in wheel cages (from 1 to 2 years of age).

### 4.1. Exercise Maintains Mitochondria–Triad Connectivity During Aging

In Zampieri et al., 2015 [212], three groups of individuals were included in the study: (a) a group of well-trained seniors (average of 70 ± 4 years of age) who routinely practiced (lifelong) sport activities, usually more than three times a week; (b) age-matched healthy sedentary seniors, performing only routine daily activities; and finally c) young men (average of 27 ± 4 years of age) physically active for three, but no more than five, times a week. The main outcome of this study was that lifelong physical exercise prevented almost completely the age-related misalignment of myofibrils, and also the disarray of EC coupling system and mitochondrial network that occurs in sedentary people (Figure 2D,E). Sedentary aging causes a loss of (i) mitochondrial volume, (ii) n./area of mitochondria, (iii) n. of triads, and finally (iv) the number of mitochondria properly coupled to triads (see Section 3.1 and Section 3.2 for more detail). In aged-trained individuals, all those parameters (i to iv) were comparable to those of active young men and approximately double than those of age-matched sedentary seniors [212]. The study also employed different experimental approaches to establish a correlation between muscle structure and improvement of function. Rescued ultrastructure of muscle fibers was accompanied by (a) an increased force (measured by assessing the maximal isometric torque produced by quadriceps muscle during extension of the knee) and (b) an improved performance in a series of functional tests (10 m walking test; short physical performance battery; timed up-and-go test; etc.). In the same paper, we also evaluated expression levels of genes involved in atrophy and hypertrophy pathways (IGF-1, Atrogin, Murf) and expression of genes related to autophagy (Beclin1, Bnip3) and ROS detoxification (NRF2, PGC1a) [212]. In parallel studies, we also showed that long-term physical exercise (a) increased the expression levels of the mitochondrial Ca^2+^ uniporter (MCU), the protein that mediates the entry of the divalent cation in the matrix [68] (b) affected the mitochondrial fission–fusion dynamics [254]; and finally (c) promoted muscle reinnervation [255].

Results collected in human biopsies were then confirmed in animal studies (and reinforced employing additional experimental techniques), where wild-type mice were aged (up to 24 months of age) in two types of cages, either equipped or non-equipped with running wheels for voluntary wheel running (VWR). Being nocturnal, mice will run on the wheel during night hours if available. The mice were divided in two groups: (a) aged sedentary mice (housed for 24 months in regular cages) and (b) aged-trained mice (housed for the first 12 months in regular cages and from 12 to 24 months of age, in cages equipped with running wheels). EM studies confirmed results collected in humans: triads and mitochondria were well preserved by exercise (n./area, volume, intracellular disposition, reciprocal association, n. of tethers) [64]. Additionally, (a) with histological analysis, we verified that atrophy was reduced in aged-trained mice; and (b) from in vivo using grip-strength test and ex vivo performing force measurements in isolated muscles electrically stimulated, we found an improved force output. As in Pietrangelo et al., 2015 [63], elevated levels of oxidative stress in muscle of aged mice were proposed to contribute to damage to membranes and organelles dedicated to Ca^2+^ handling and aerobic ATP production (see Section 3.1 and Section 3.3 for additional detail). We also investigated levels of oxidative stress in mice exercised in wheel cages and found that exercise normalized oxidative stress (compared to sedentary mice in which oxidative stress was elevated), while normalizing internal ultrastructure of fibers [64].

### 4.2. Exercise Reduces Tubular Aggregates (TAs) and Maintains SOCE Function

EDL muscles of mice kept is standard cages (i.e., that did not have access to running wheels) presented accumulation of TAs, and loss of SOCE function due to reduced presence of the two structural elements needed for the assembly of functional CEUs: SR stacks and elongated TTs at the I band [181] (see Section 3.3 for additional detail). We also demonstrated that TAs contain STIM1, ORAI1, and CASQ1 apparently trapped in a dysfunctional compartment [134,181]. The accumulation of TAs, the presence of membrane elements needed for assembly of CEUs, and the function of SOCE were then studied in mice that had access to running wheels for 12 months (from 1 to 2 years of age). The results of this effort demonstrated that long-term VWR (1 year) was quite effective in (a) reducing formation of TAs (outlined in blue in Figure 2F), (b) preventing loss of SR stacks and TTs at the I band (inset in Figure 2F), and (c) rescuing functional SOCE, as shown by gain-of-function during a fatigue protocol in the presence of external Ca^2+^ (which could be blocked in 0 Ca^2+^ or by the presence of an SOCE inhibitor) [181]. The interpretation of these findings would be that, as SOCE is a mechanism that normally is recruited during muscle activity (which induces fatigue and SR depletion), in sedentary people/mice, it would be rarely activated, leading to complete inactivation of the mechanism (reduced presence of CEUs and improper accumulation of STIM1, ORAI1, and CASQ1 in TAs), while in muscle that is regularly exercised, SOCE will remain active throughout life (presence of CEUs, reduced formation of TAs, and improved SOCE function).

## 5. Studies in Different Experimental Models Indicate That Age-like Modifications Are Induced by Inactivity and Are Reversible

In Section 3, we described how sedentary aging results in disarray of the mitochondrial network and sarcotubular system. One aspect that also needs to be taken into consideration (besides increasing age) is the progressive reduction in activity associated with aging, both in humans (due to a sedentary lifestyle) and in animals, which are usually kept in small cages (which restrict their daily activities). Hence, it remains to be determined whether the alterations described in Section 3 are induced by aging *per se*, or whether the reduced muscle activity associated with sedentary aging plays a central role in those modifications. We are currently trying to give an answer to this relevant question by studying short-term denervation and immobilization in adult animals (rats and mice). Also, we are trying to determine whether the remodeling of the mitochondrial network and the sarcotubular system due to inactivity is reversible.

### 5.1. Effect of Denervation and Short-Term Immobilization

Muscle fibers contract in response to action potentials delivered by motor neurons at the NMJ, i.e., the site of contact between the nervous system and muscle cells. When communication at the NMJ is interrupted, muscle fibers become *denervated* [256,257,258,259]. Denervation may be caused by traumatic events (spinal cord injuries or lesion of peripheral nerves) or by neurodegenerative diseases. Notably, denervation of motor units also occurs spontaneously during aging, especially in sedentary individuals. Indeed, denervation and consequent loss of motor units seem to be an important contributing factor to aged-related atrophy and sarcopenia, with fast-twitch fibers (type II) being more affected by denervation than slow-twitch fibers (type I) [200,260,261,262,263,264,265].

The effect of denervation on muscle fibers, i.e., the great reduction in fiber diameter (atrophy) and the complete disruption of contractile elements, is well documented in the literature [266,267,268,269,270]. Less is known about the effect of denervation on the EC coupling system [271], and, to my knowledge, the effect of denervation on mitochondria has been completely overlooked. Between 2004 and 2019, we have studied the effect of long- and short-term denervation on muscle fibers in human biopsies from spinal cord injury (SCI) patients (with complete lesion of the conus cauda) and in muscles of rabbits and rats [64,272,273,274,275]. In human biopsies of SCI patients, in which muscles were denervated for extended periods of time (from 1 year up to several years), fibers were compromised to the point in which myofibrils and striation were almost completely lost. In these severely compromised fibers, (a) mitochondria were few and often clustered in small groups; and (b) some triads were still present, even if their morphology was abnormal [276,277]. It was argued that the presence of these residual elements of the EC coupling machinery could explain why, even after years of denervation, the denervated fibers could be rescued by functional electrical stimulation (FES, see below) [273,275,276,277,278].

While long-term denervation studies gave important insights, the analysis of the mitochondrial network and of the EC coupling system in muscles denervated for shorter times was more informative about the time-course of changes leading to the disarray of these systems. We first studied the effects of denervation in muscles of rats and rabbits (3–8 months of denervation), in which contractile elements are partially misaligned, but still present: in this model, mitochondria are already displaced from their I band position next to triads, becoming longitudinally orientated, with many of them migrating toward the A band [274,275]. This observation allowed a first important conclusion: the misplacement of mitochondria from their correct positioning at the I band precedes the complete loss of striation. Later, studies in mice and rats denervated for much shorter periods (3 to 15 days) showed that the displacement of mitochondria from their correct transversal position at the I band is a quite fast phenomenon [64]: after 14–15 days of denervation, respectively, in mice and rats (in which misalignment of myofibrils is quite limited), several mitochondria are already longitudinally oriented. Minor movement of mitochondria was even detected in muscle of mice denervated for only 3 days [64]. Displacement of mitochondria from the correct I band position was accompanied by (a) a reduction in the number of mitochondria–triad couples, also due to a decreased number of triads (see below); and (b) altered mitochondrial Ca^2+^ uptake.

The analysis of the EC coupling system in muscles denervated for short times was also informative. The de-modeling of the TT network, from exclusively transversal to progressively more longitudinal, is a process that interestingly mimics (but in a reverse-mode) the maturation process of the TT system, which starts to develop longitudinally before becoming transversal [42,43,279]. The longitudinal TTs in denervated fibers are still connected to the extracellular space, as shown by positive staining with ferrocyanide applied externally [275]. Loss of proper architecture of TTs results in triads that lose proper orientation (i.e., becoming oblique or longitudinal), display altered morphology, and progressively disappear as their number is greatly reduced. Notably, the changes affecting the sarcotubular system in short-denervated fibers closely resemble those in fibers of elderly individuals (see Section 3.1 for additional detail) [63,213]. Another important aspect deserves to be underlined: during post-natal maturation of muscle fibers, the EC coupling system becomes transversal before the mitochondrial network [33], while in aging and short-term denervation, the mitochondria seem to lose proper disposition before the sarcotubular system starts to become longitudinal [63,64]. These two observations together suggest that mitochondrial positioning seems less stable than that of TT and SR (which seem to be better anchored to the internal cytoskeleton of fibers and to cross-striation), at least in the first phase of muscle inactivity.

To determine if the effect of denervation is caused by lack of contact with the nerve or by muscle being inactive, we recently generated a model of short-term inactivity, obtained by unilateral six-day immobilization of a hind limb in mice. The results of these experiments (unpublished; Pietrangelo et al., submitted for publication and [280]) indicate that (a) even short-term inactivity causes remodeling of the mitochondrial network and of membrane systems involved in Ca^2+^ handling; (b) the effects of short-term immobilization mimics quite closely the effect of short-term denervation and aging [64], strongly suggesting that muscle inactivity is the leading cause of the internal remodeling of fibers during aging.

### 5.2. Functional Electrical Stimulation (FES), Reinnervation, and Exercise Reverse the Effect of Denervation and Short-Term Immobilization

Functional electrical stimulation (FES) is a form of treatment to send electric currents to nerves and muscles [281,282,283]. FES is used in some applications to complement exercise and help restore muscle function, while in case of denervation, it may be used to keep/restore functions of muscles no longer connected to the nervous system. For about 15 years, starting in 2004, we collaborated with the group of Dr. Helmut Kern in Vienna and studied the effect of FES on muscle in different projects: (a) in patients with spinal cord injury (SCI) to restore muscle structure and mass [272,276,277]; (b) in animal models to counteract the effect of surgical denervation [273,274,275]; (c) as a supportive measure to improve muscle function in elderly individuals [284]; and (d) as a therapy in a patient affected by central core disease (CCD), a rare myopathy of genetic origin that causes mitochondrial damage and muscle weakness [285].

Muscles of patients who suffered complete SCI were subjected to FES for prolonged periods (several years), although stimulation was started when muscle alterations due to denervation were already quite severe (one year or more after injury [272,276]). We performed EM analysis of biopsies from those patients and found FES-induced restoration of denervated muscles [276,277]. Interestingly, beside FES-induced rescue of contractile elements and muscle mass, we also found a surprising restoration of the membrane elements that mediate EC coupling (i.e., triads), which were placed in the correct position at the I-A band transition of sarcomeres. Retrospective analysis of EM micrographs performed a few years later also revealed a rescue of mitochondria (unpublished observation). FES was also effective in restoring internal structure of fibers in denervated rabbits [273] and in a CCD patient [285]. The mechanism underlying the rescue of muscle fiber structure and function by FES in the absence of normal innervation remains unclear. Some of the published findings may explain why long-term denervated muscle responds to FES: (a) we showed that EC coupling units (i.e., triads), while deformed, are still present in muscle fibers of SCI patients even after several years of denervation [276,277,278]; (b) in rats, after months of denervation injury, skeletal fibers were still able to be excited, i.e., to carry an action potential and contained triads with TT connected to external membrane [275]; finally, (c) we reported the presence of some regenerating fibers (expressing embryonic myosin) and some atrophy-resistant fibers in long-term denervated muscle [286,287].

The interest of the results collected using FES in SCI, CCD, and healthy aging for the present review is the fact that the modifications caused by muscle inactivity to mitochondria and the sarcotubular system appear to be reversible. This important aspect has been further investigated in more recent projects:

a. In Pietrangelo et al., 2019 [64], we showed that reinnervation (occurring spontaneously) in rat muscles that were previously denervated by nerve crash completely restored the position of mitochondria at the I band and the transversal organization of the TT network, which was previously compromised by the lack of muscle activity (caused by lack of nerve impulses due to denervation).
b. Two weeks or treadmill rehabilitation (3–4 times a week) in mice that were previously subjected to hind limb unilateral immobilization for six days (by casting) completely rescued the proper intracellular organization of mitochondria, triads, and CEUs (Pietrangelo et al., submitted for publication and [280]).

## 6. Summary and Final Remarks

Skeletal muscle represents roughly 40–60% of the body weight of a healthy adult individual. The relative percentage of body weight occupied by skeletal muscle will change during life and will vary in different individuals depending on several factors: in general, it will be higher in those who exercise/train regularly (and have a lower percentage of fat mass), and it would be lower and decrease progressively in sedentary people, especially during aging. Indeed, the age-related loss of skeletal muscle mass, known as *sarcopenia*, is significantly more pronounced in those who age with a sedentary lifestyle, compared to those who exercise regularly while aging [205,288,289,290].

Adult and healthy skeletal muscle fibers are multinucleated cells, beautifully designed to produce efficient force while burning energy. However, to work properly, skeletal fibers need a specific arrangement of the intracellular organelles and membranes dedicated to force production (myofibrils), aerobic ATP production (mitochondria), and Ca^2+^ handling (triads and CEUs), which is achieved during a process of post-natal maturation that may take months to years depending on the species we are taking into consideration (e.g., mice/rats vs. humans).

Skeletal muscle fibers, beside generating force and movements of the skeleton, also have other important functions, ranging from being an important endocrine organ (i.e., when active, it secretes important myokines that have trophic effects on several other organs) [291,292,293] to being able to control the metabolic balance and the thermoregulation of individuals. Metabolic balance and thermoregulation are two interconnected concepts because thermoregulation usually uses about 50% of the metabolic cost at rest. Each individual has a rest metabolic rate that depends on several factors: percentage of lean body weight, sex, age, genetic factors (which control the relative percentage of fast vs. slow-twitch fibers in skeletal muscle), etc. In general, the only way that an individual has to consume more calories is to increase daily activities (or to decrease temperature of the living environment, to use energy for thermoregulation). The reason why muscle is so important for the metabolic rate is because muscle fibers, the multinucleated cells that constitute muscles, contain the majority of mitochondria of the entire body, with mitochondria being the organelles that consume oxygen to burn the caloric intake.

Proper mitochondrial function (hence efficient production of ATP by mitochondria) is ensured by a normal number and volume of mitochondria. Sedentary aging, or simply inactivity in adult age, causes both loss of muscle mass and loss of mitochondria, impairing the capability of muscle to burn the daily caloric intake [294,295,296]. Worth noting is also the fact that the daily caloric intake is often in excess in sedentary humans, especially in more industrialized countries, and especially during aging.

In this review, we focused our attention to an aspect that, we believe, should also be taken into consideration: the fact that, to function properly, mitochondria need to be correctly positioned inside muscle fibers (i.e., at the I band of striated fibers, close to the membrane systems that controls Ca^2+^ handling during EC coupling and SOCE), and that this position is finely controlled by muscle activity. We have shown that only few days of inactivity are sufficient to move some mitochondria away from the correct position [64]. The importance of mitochondria being at the I band is likely related to the fact that in that position mitochondria are placed next to Ca^2+^ release sites (the triads, or CRUs), but also in the same exact region of the SR that is involved in the exercise-dependent remodeling that leads to the assembly of CEUs, the junctions that boost Ca^2+^ entry from the extracellular space. We have also shown that sedentary aging and inactivity also affects the sarcotubular system in a way that proper Ca^2+^ signaling can be impaired: reduction in the number of triads (which impairs EC coupling) and complete loss of CEUs (resulting in dysfunctional SOCE). Though, it is important to underline that misplacement of mitochondria and loss of CRUs and CEUs appear to be a reversible process, as they can be prevented or rescued by muscle activity/exercise.

**Figure 3:**
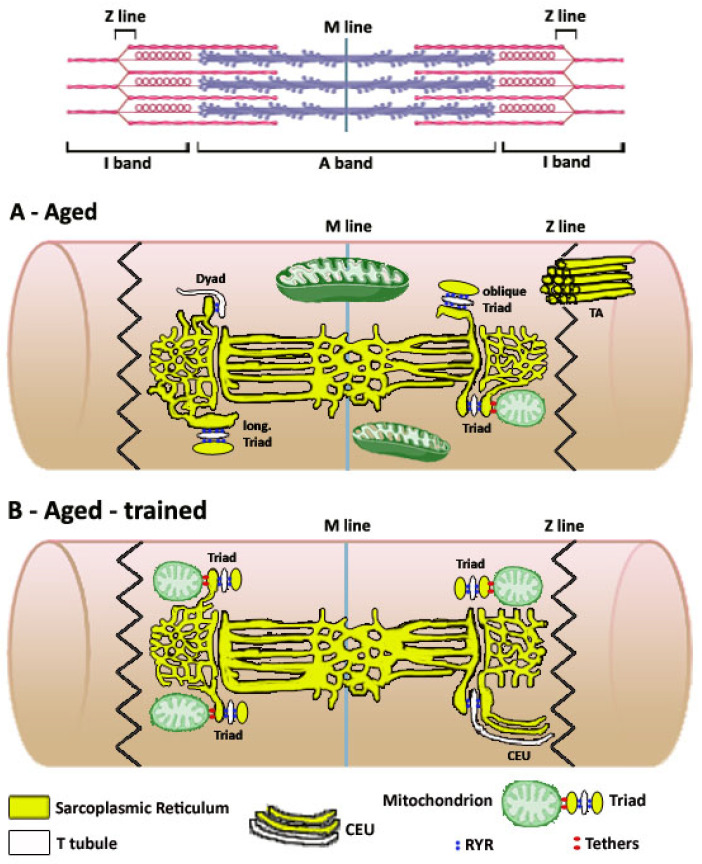
Model summarizing the alterations caused by ageing (panel (**A**)) and the positive effects of exercise (panel (**B**)). Proper position of organelles (triads, mitochondria, and CEUs) in skeletal muscle fibers is dictated by the cross-striation generated by I and A bands of sarcomeres (top drawing). (**A**) In skeletal muscle fibers of aged mice, some mitochondria are misplaced at the A band, some triads become oblique or longitudinally oriented, while the SR may form TAs. (**B**) Exercise maintains (or rescues) the correct position of mitochondria and triads at the I band and reduces the formation of TAs while promoting the assembly of CEUs.

Some aspects, however, still require additional investigation. What at the moment remains to be determined is whether the disarray of the mitochondrial network and sarcotubular system occurring during sedentary aging (Figure 3A) can be reversed after extended periods of inactivity. Indeed, in our experiments in human biopsies and aging mice, we only showed that this phenomenon can be prevented by regular exercise throughout life (Figure 3B), but we did not demonstrate that the internal structure of fibers can be rescued in aged individuals (or mice) who restart exercising late in life, possibly after many years (or months in mice) of sedentary life. This gap of knowledge opens a potentially interesting new line of investigation for our future research.

## Data Availability

No new data were created or analyzed in this study.

## Abbreviations

The following abbreviations are used in this manuscript:

CRU	Calcium release unit
CEU	Calcium entry unit
EM	Electron microscopy
EC	Excitation–contraction
SR	Sarcoplasmic reticulum
SOCE	Store-operated Ca^2+^ entry
TA	Tubular aggregate
TT	Transverse tubule

## References

[1] Rasmussen H., Jensen P., Lake W., Goodman D.B. (1976). Calcium Ion as Second Messenger. Clin. Endocrinol..

[2] Gerke V., Creutz C.E., Moss S.E. (2005). Annexins: Linking Ca^2+^ Signalling to Membrane Dynamics. Nat. Rev. Mol. Cell Biol..

[3] Endo M. (2006). Calcium Ion as a Second Messenger With Special Reference to Excitation-Contraction Coupling. J. Pharmacol. Sci..

[4] Dolmetsch R. (2003). Excitation-Transcription Coupling: Signaling by Ion Channels to the Nucleus. Sci. STKE.

[5] Clapham D.E. (2007). Calcium Signaling. Cell.

[6] Marchi S., Patergnani S., Missiroli S., Morciano G., Rimessi A., Wieckowski M.R., Giorgi C., Pinton P. (2018). Mitochondrial and Endoplasmic Reticulum Calcium Homeostasis and Cell Death. Cell Calcium.

[7] Shkryl V.M., Shirokova N. (2006). Transfer and Tunneling of Ca^2+^ from Sarcoplasmic Reticulum to Mitochondria in Skeletal Muscle. J. Biol. Chem..

[8] Rudolf R., Mongillo M., Magalhães P.J., Pozzan T. (2004). In Vivo Monitoring of Ca^2+^ Uptake into Mitochondria of Mouse Skeletal Muscle during Contraction. J. Cell Biol..

[9] Bolaños P., Calderón J.C. (2022). Excitation-Contraction Coupling in Mammalian Skeletal Muscle: Blending Old and Last-Decade Research. Front. Physiol..

[10] Gherardi G., Monticelli H., Rizzuto R., Mammucari C. (2020). The Mitochondrial Ca^2+^ Uptake and the Fine-Tuning of Aerobic Metabolism. Front. Physiol..

[11] Bolaños P., Guillen A., Rojas H., Boncompagni S., Caputo C. (2007). The Use of CalciumOrange-5N as a Specific Marker of Mitochondrial Ca^2+^ in Mouse Skeletal Muscle Fibers. Pflug. Arch..

[12] Rossi A.E., Boncompagni S., Dirksen R.T. (2009). Sarcoplasmic Reticulum-Mitochondrial Symbiosis. Exerc. Sport. Sci. Rev..

[13] Eisner V., Csordás G., Hajnóczky G. (2013). Interactions between Sarco-Endoplasmic Reticulum and Mitochondria in Cardiac and Skeletal Muscle—Pivotal Roles in Ca^2+^ and Reactive Oxygen Species Signaling. J. Cell Sci..

[14] Csordás G., Hajnóczky G. (2009). SR/ER-Mitochondrial Local Communication: Calcium and ROS. Biochim. Biophys. Acta.

[15] Sandow A. (1952). Excitation-Contraction Coupling in Muscular Response. Yale J. Biol. Med..

[16] Sandow A., Taylor S.R., Preiser H. (1965). Role of the Action Potential in Excitation-Contraction Coupling. Fed. Proc..

[17] Schneider M.F., Rodney G.G., Ward C.W. (2004). Local Ca^2+^ Release Events in Skeletal Muscle. J. Muscle Res. Cell Motil..

[18] Franzini-Armstrong C., Protasi F. (1997). Ryanodine Receptors of Striated Muscles: A Complex Channel Capable of Multiple Interactions. Physiol. Rev..

[19] Calderón J.C., Bolaños P., Caputo C. (2014). The Excitation–Contraction Coupling Mechanism in Skeletal Muscle. Biophys. Rev..

[20] Stokes D.L., Wagenknecht T. (2000). Calcium Transport across the Sarcoplasmic Reticulum: Structure and Function of Ca^2+^-ATPase and the Ryanodine Receptor. Eur. J. Biochem..

[21] Hernández-Ochoa E.O., Pratt S.J.P., Lovering R.M., Schneider M.F. (2015). Critical Role of Intracellular RyR1 Calcium Release Channels in Skeletal Muscle Function and Disease. Front. Physiol..

[22] Putney J.W. (1986). A Model for Receptor-Regulated Calcium Entry. Cell Calcium.

[23] Parekh A.B., Penner R. (1997). Store Depletion and Calcium Influx. Physiol. Rev..

[24] Launikonis B.S., Ríos E. (2007). Store-operated Ca^2+^ Entry during Intracellular Ca^2+^ Release in Mammalian Skeletal Muscle. J. Physiol..

[25] Launikonis B.S., Barnes M., Stephenson D.G. (2003). Identification of the Coupling between Skeletal Muscle Store-Operated Ca^2+^ Entry and the Inositol Trisphosphate Receptor. Proc. Natl. Acad. Sci. USA.

[26] Kurebayashi N., Ogawa Y. (2001). Depletion of Ca^2+^ in the Sarcoplasmic Reticulum Stimulates Ca^2+^ Entry into Mouse Skeletal Muscle Fibres. J. Physiol..

[27] Dirksen R.T. (2009). Checking Your SOCCs and Feet: The Molecular Mechanisms of Ca^2+^ Entry in Skeletal Muscle. J. Physiol..

[28] Michelucci A., García-Castañeda M., Boncompagni S., Dirksen R.T. (2018). Role of STIM1/ORAI1-Mediated Store-Operated Ca^2+^ Entry in Skeletal Muscle Physiology and Disease. Cell Calcium.

[29] Protasi F., Girolami B., Serano M., Pietrangelo L., Paolini C. (2022). Ablation of Calsequestrin-1, Ca^2+^ Unbalance, and Susceptibility to Heat Stroke. Front. Physiol..

[30] Sztretye M., Geyer N., Vincze J., Al-Gaadi D., Oláh T., Szentesi P., Kis G., Antal M., Balatoni I., Csernoch L. (2017). SOCE Is Important for Maintaining Sarcoplasmic Calcium Content and Release in Skeletal Muscle Fibers. Biophys. J..

[31] Wei-Lapierre L., Carrell E.M., Boncompagni S., Protasi F., Dirksen R.T. (2013). Orai1-Dependent Calcium Entry Promotes Skeletal Muscle Growth and Limits Fatigue. Nat. Commun..

[32] Lilliu E., Koenig S., Koenig X., Frieden M. (2021). Store-Operated Calcium Entry in Skeletal Muscle: What Makes It Different?. Cells.

[33] Boncompagni S., Rossi A.E., Micaroni M., Beznoussenko G.V., Polishchuk R.S., Dirksen R.T., Protasi F. (2009). Mitochondria Are Linked to Calcium Stores in Striated Muscle by Developmentally Regulated Tethering Structures. Mol. Biol. Cell.

[34] Rossi A.E., Boncompagni S., Wei L., Protasi F., Dirksen R.T. (2011). Differential Impact of Mitochondrial Positioning on Mitochondrial Ca^2+^ Uptake and Ca^2+^ Spark Suppression in Skeletal Muscle. Am. J. Physiol.-Cell Physiol..

[35] Franzini-Armstrong C., Boncompagni S. (2011). The Evolution of the Mitochondria-to-Calcium Release Units Relationship in Vertebrate Skeletal Muscles. J. Biomed. Biotechnol..

[36] Marchioretti C., Zanetti G., Pirazzini M., Gherardi G., Nogara L., Andreotti R., Martini P., Marcucci L., Canato M., Nath S.R. (2023). Defective Excitation-Contraction Coupling and Mitochondrial Respiration Precede Mitochondrial Ca^2+^ Accumulation in Spinobulbar Muscular Atrophy Skeletal Muscle. Nat. Commun..

[37] Hatano A., Okada J., Washio T., Hisada T., Sugiura S. (2013). Mitochondrial Colocalization with Ca^2+^ Release Sites Is Crucial to Cardiac Metabolism. Biophys. J..

[38] Santo-Domingo J., Demaurex N. (2010). Calcium Uptake Mechanisms of Mitochondria. Biochim. Biophys. Acta.

[39] Franzini-Armstrong C. (1970). Studies of the triad. J. Cell Biol..

[40] Franzini-Armstrong C., Jorgensen A.O. (1994). Structure and Development of E-C Coupling Units in Skeletal Muscle. Annu. Rev. Physiol..

[41] Flucher B.E., Andrews S.B., Daniels M.P. (1994). Molecular Organization of Transverse Tubule/Sarcoplasmic Reticulum Junctions during Development of Excitation-Contraction Coupling in Skeletal Muscle. Mol. Biol. Cell.

[42] Takekura H., Bennett L., Tanabe T., Beam K.G., Franzini-Armstrong C. (1994). Restoration of Junctional Tetrads in Dysgenic Myotubes by Dihydropyridine Receptor CDNA. Biophys. J..

[43] Flucher B.E., Franzini-Armstrong C. (1996). Formation of Junctions Involved in Excitation-Contraction Coupling in Skeletal and Cardiac Muscle. Proc. Natl. Acad. Sci. USA.

[44] Boncompagni S., Michelucci A., Pietrangelo L., Dirksen R.T., Protasi F. (2017). Exercise-Dependent Formation of New Junctions That Promote STIM1-Orai1 Assembly in Skeletal Muscle. Sci. Rep..

[45] Michelucci A., Boncompagni S., Pietrangelo L., Takano T., Protasi F., Dirksen R.T. (2020). Pre-Assembled Ca^2+^ Entry Units and Constitutively Active Ca^2+^ Entry in Skeletal Muscle of Calsequestrin-1 Knockout Mice. J. Gen. Physiol..

[46] Michelucci A., Boncompagni S., Pietrangelo L., García-Castañeda M., Takano T., Malik S., Dirksen R.T., Protasi F. (2019). Transverse Tubule Remodeling Enhances Orai1-Dependent Ca^2+^ Entry in Skeletal Muscle. Elife.

[47] Michelucci A., Pietrangelo L., Rastelli G., Protasi F., Dirksen R.T., Boncompagni S. (2022). Constitutive Assembly of Ca^2+^ Entry Units in Soleus Muscle from Calsequestrin Knockout Mice. J. Gen. Physiol..

[48] Protasi F., Pietrangelo L., Boncompagni S. (2021). Improper Remodeling of Organelles Deputed to Ca^2+^ Handling and Aerobic ATP Production Underlies Muscle Dysfunction in Ageing. Int. J. Mol. Sci..

[49] Protasi F., Girolami B., Roccabianca S., Rossi D. (2023). Store-Operated Calcium Entry: From Physiology to Tubular Aggregate Myopathy. Curr. Opin. Pharmacol..

[50] Ebashi S., Endo M. (1968). Calcium Ion and Muscle Contraction. Prog. Biophys. Mol. Biol..

[51] Lymn R.W., Taylor E.W. (1971). Mechanism of Adenosine Triphosphate Hydrolysis by Actomyosin. Biochemistry.

[52] Castillo J.P., Rui H., Basilio D., Das A., Roux B., Latorre R., Bezanilla F., Holmgren M. (2015). Mechanism of Potassium Ion Uptake by the Na^+^/K^+^-ATPase. Nat. Commun..

[53] Sweeney H.L. (1994). The Importance of the Creatine Kinase Reaction: The Concept of Metabolic Capacitance. Med. Sci. Sports Exerc..

[54] Bonora M., Patergnani S., Rimessi A., De Marchi E., Suski J.M., Bononi A., Giorgi C., Marchi S., Missiroli S., Poletti F. (2012). ATP Synthesis and Storage. Purinergic Signal..

[55] Farrell P.A., Joyner M.J., Caiozzo V.J. (2012). ACSM’s Advanced Exercise Physiology.

[56] Territo P.R., Mootha V.K., French S.A., Balaban R.S. (2000). Ca^2+^ Activation of Heart Mitochondrial Oxidative Phosphorylation: Role of the F_0_/F_1_-ATPase. Am. J. Physiol.-Cell Physiol..

[57] McMillin J.B., Madden M.C. (1989). The Role of Calcium in the Control of Respiration by Muscle Mitochondria. Med. Sci. Sports Exerc..

[58] Hansford R.G. (1987). Relation between Cytosolic Free Ca^2+^ Concentration and the Control of Pyruvate Dehydrogenase in Isolated Cardiac Myocytes. Biochem. J..

[59] Isaeva E.V., Shkryl V.M., Shirokova N. (2005). Mitochondrial Redox State and Ca^2+^ Sparks in Permeabilized Mammalian Skeletal Muscle. J. Physiol..

[60] Isaevaand E.V., Shirokova N. (2003). Metabolic Regulation of Ca^2+^ Release in Permeabilized Mammalian Skeletal Muscle Fibres. J. Physiol..

[61] Hansford R.G. (1994). Role of Calcium in Respiratory Control. Med. Sci. Sports Exerc..

[62] Gillis J.M. (1997). Inhibition of Mitochondrial Calcium Uptake Slows down Relaxation in Mitochondria-Rich Skeletal Muscles. J. Muscle Res. Cell Motil..

[63] Pietrangelo L., D’Incecco A., Ainbinder A., Michelucci A., Kern H., Dirksen R.T., Boncompagni S., Protasi F. (2015). Age-Dependent Uncoupling of Mitochondria from Ca^2+^ Release Units in Skeletal Muscle. Oncotarget.

[64] Pietrangelo L., Michelucci A., Ambrogini P., Sartini S., Guarnier F.A., Fusella A., Zamparo I., Mammucari C., Protasi F., Boncompagni S. (2019). Muscle Activity Prevents the Uncoupling of Mitochondria from Ca^2+^ Release Units Induced by Ageing and Disuse. Arch. Biochem. Biophys..

[65] Buntinas L., Gunter K.K., Sparagna G.C., Gunter T.E. (2001). The Rapid Mode of Calcium Uptake into Heart Mitochondria (RaM): Comparison to RaM in Liver Mitochondria. Biochim. Biophys. Acta (BBA)—Bioenerg..

[66] Beutner G., Sharma V.K., Giovannucci D.R., Yule D.I., Sheu S.-S. (2001). Identification of a Ryanodine Receptor in Rat Heart Mitochondria. J. Biol. Chem..

[67] Gunter K.K., Gunter T.E. (1994). Transport of Calcium by Mitochondria. J. Bioenerg. Biomembr..

[68] De Stefani D., Raffaello A., Teardo E., Szabò I., Rizzuto R. (2011). A Forty-Kilodalton Protein of the Inner Membrane Is the Mitochondrial Calcium Uniporter. Nature.

[69] Baughman J.M., Perocchi F., Girgis H.S., Plovanich M., Belcher-Timme C.A., Sancak Y., Bao X.R., Strittmatter L., Goldberger O., Bogorad R.L. (2011). Integrative Genomics Identifies MCU as an Essential Component of the Mitochondrial Calcium Uniporter. Nature.

[70] Pan X., Liu J., Nguyen T., Liu C., Sun J., Teng Y., Fergusson M.M., Rovira I.I., Allen M., Springer D.A. (2013). The Physiological Role of Mitochondrial Calcium Revealed by Mice Lacking the Mitochondrial Calcium Uniporter. Nat. Cell Biol..

[71] Sembrowich W.L., Quintinskie J.J., Li G. (1985). Calcium Uptake in Mitochondria from Different Skeletal Muscle Types. J. Appl. Physiol..

[72] Mannella C.A., Buttle K., Rath B.K., Marko M. (1998). Electron Microscopic Tomography of Rat-liver Mitochondria and Their Interactions with the Endoplasmic Reticulum. BioFactors.

[73] Csordás G., Renken C., Várnai P., Walter L., Weaver D., Buttle K.F., Balla T., Mannella C.A., Hajnóczky G. (2006). Structural and Functional Features and Significance of the Physical Linkage Between ER and Mitochondria. J. Cell Biol..

[74] Sharma V.K., Ramesh V., Franzini-Armstrong C., Sheu S.-S. (2000). Transport of Ca^2+^ from Sarcoplasmic Reticulum to Mitochondria in Rat Ventricular Myocytes. J. Bioenergetics Biomembr..

[75] Ogata T., Yamasaki Y. (1985). Scanning Electron-Microscopic Studies on the Three-Dimensional Structure of Mitochondria in the Mammalian Red, White and Intermediate Muscle Fibers. Cell Tissue Res..

[76] Ainbinder A., Boncompagni S., Protasi F., Dirksen R.T. (2015). Role of Mitofusin-2 in Mitochondrial Localization and Calcium Uptake in Skeletal Muscle. Cell Calcium.

[77] Schneider M.F. (1994). Control of Calcium Release in Functioning Skeletal Muscle Fibers. Annu. Rev. Physiol..

[78] Protasi F. (2002). Structural Interaction between RYRs and DHPRs in Calcium Release Units of Cardiac and Skeletal Muscle Cells. Front. Biosci..

[79] Ebashi S. (1991). Excitation-Contraction Coupling and the Mechanism of Muscle Contraction. Annu. Rev. Physiol..

[80] Wray S., Ravens U., Verkhratsky A., Eisner D. (2004). Two Centuries of Excitation–Contraction Coupling. Cell Calcium.

[81] Rassier D.E. (2017). Sarcomere Mechanics in Striated Muscles: From Molecules to Sarcomeres to Cells. Am. J. Physiol. Cell Physiol..

[82] Herzog W. (2022). What Can We Learn from Single Sarcomere and Myofibril Preparations?. Front. Physiol..

[83] Heilbrunn L.V., Wiercinski F.J. (1947). The Action of Various Cations on Muscle Protoplasm. J. Cell Comp. Physiol..

[84] Niedergerke R. (1955). The Staircase Phenomenon in the Frog’s Ventricle and the Action of Calcium. J. Physiol..

[85] Hasselbach W., Makinose M. (1961). The Calcium Pump of the “Relaxing Granules” of Muscle and Its Dependence on ATP-Splitting. Biochem. Z..

[86] Hasselbach W. (1964). Relaxation and the sarcotubular calcium pump. Fed. Proc..

[87] Hasselbach W., Oetliker H. (1983). Energetics and Electrogenicity of the Sarcoplasmic Reticulum Calcium Pump. Annu. Rev. Physiol..

[88] Schneider M.F., Chandler W.K. (1973). Voltage Dependent Charge Movement in Skeletal Muscle: A Possible Step in Excitation–Contraction Coupling. Nature.

[89] Ríos E., Pizarro G. (1991). Voltage Sensor of Excitation-Contraction Coupling in Skeletal Muscle. Physiol. Rev..

[90] Ríos E., Ma J., González A. (1991). The Mechanical Hypothesis of Excitation—Contraction (EC) Coupling in Skeletal Muscle. J. Muscle Res. Cell Motil..

[91] Ríos E., Karhanek M., Ma J., González A. (1993). An Allosteric Model of the Molecular Interactions of Excitation-Contraction Coupling in Skeletal Muscle. J. Gen. Physiol..

[92] Orkand R.K., Niedergerke R. (1964). Heart Action Potential: Dependence on External Calcium and Sodium Ions. Science.

[93] Näbauer M., Callewaert G., Cleemann L., Morad M. (1989). Regulation of Calcium Release Is Gated by Calcium Current, Not Gating Charge, in Cardiac Myocytes. Science.

[94] Fabiato A. (1983). Calcium-Induced Release of Calcium from the Cardiac Sarcoplasmic Reticulum. Am. J. Physiol.-Cell Physiol..

[95] Fabiato A. (1985). Simulated Calcium Current Can Both Cause Calcium Loading in and Trigger Calcium Release from the Sarcoplasmic Reticulum of a Skinned Canine Cardiac Purkinje Cell. J. Gen. Physiol..

[96] Cannell M., Cheng H., Lederer W. (1995). The Control of Calcium Release in Heart Muscle. Science.

[97] Collier M.L., Thomas A.P., Berlin J.R. (1999). Relationship between L-type Ca^2+^ Current and Unitary Sarcoplasmic Reticulum Ca^2+^ Release Events in Rat Ventricular Myocytes. J. Physiol..

[98] Collier M.L., Ji G., Wang Y.-X., Kotlikoff M.I. (2000). Calcium-Induced Calcium Release in Smooth Muscle. J. Gen. Physiol..

[99] Bers D.M. (2002). Cardiac Excitation–Contraction Coupling. Nature.

[100] Rios E., Brum G. (1987). Involvement of Dihydropyridine Receptors in Excitation–Contraction Coupling in Skeletal Muscle. Nature.

[101] Mikami A., Imoto K., Tanabe T., Niidome T., Mori Y., Takeshima H., Narumiya S., Numa S. (1989). Primary Structure and Functional Expression of the Cardiac Dihydropyridine-Sensitive Calcium Channel. Nature.

[102] Tanabe T., Takeshima H., Mikami A., Flockerzi V., Takahashi H., Kangawa K., Kojima M., Matsuo H., Hirose T., Numa S. (1987). Primary Structure of the Receptor for Calcium Channel Blockers from Skeletal Muscle. Nature.

[103] Tanabe T., Beam K.G., Adams B.A., Niidome T., Numa S. (1990). Regions of the Skeletal Muscle Dihydropyridine Receptor Critical for Excitation–Contraction Coupling. Nature.

[104] Tanabe T., Beam K.G., Powell J.A., Numa S. (1988). Restoration of Excitation—Contraction Coupling and Slow Calcium Current in Dysgenic Muscle by Dihydropyridine Receptor Complementary DNA. Nature.

[105] Sorrentino V., Volpe P. (1993). Ryanodine Receptors: How Many, Where and Why?. Trends Pharmacol. Sci..

[106] Giannini G., Sorrentino V. (1995). Molecular Structure and Tissue Distribution of Ryanodine Receptors Calcium Channels. Med. Res. Rev..

[107] Sorrentino V. (1995). The Ryanodine Receptor Family of Intracellular Calcium Release Channels. Adv. Pharmacol..

[108] Zorzato F., Fujii J., Otsu K., Phillips M., Green N.M., Lai F.A., Meissner G., MacLennan D.H. (1990). Molecular Cloning of CDNA Encoding Human and Rabbit Forms of the Ca^2+^ Release Channel (Ryanodine Receptor) of Skeletal Muscle Sarcoplasmic Reticulum. J. Biol. Chem..

[109] Takeshima H., Nishimura S., Matsumoto T., Ishida H., Kangawa K., Minamino N., Matsuo H., Ueda M., Hanaoka M., Hirose T. (1989). Primary Structure and Expression from Complementary DNA of Skeletal Muscle Ryanodine Receptor. Nature.

[110] Nakai J., Sekiguchi N., Rando T.A., Allen P.D., Beam K.G. (1998). Two Regions of the Ryanodine Receptor Involved in Coupling Withl-Type Ca^2+^ Channels. J. Biol. Chem..

[111] Block B.A., Imagawa T., Campbell K.P., Franzini-Armstrong C. (1988). Structural Evidence for Direct Interaction between the Molecular Components of the Transverse Tubule/Sarcoplasmic Reticulum Junction in Skeletal Muscle. J. Cell Biol..

[112] Protasi F., Franzini-Armstrong C., Flucher B.E. (1997). Coordinated Incorporation of Skeletal Muscle Dihydropyridine Receptors and Ryanodine Receptors in Peripheral Couplings of BC_3_H1 Cells. J. Cell Biol..

[113] Protasi F., Franzini-Armstrong C., Allen P.D. (1998). Role of Ryanodine Receptors in the Assembly of Calcium Release Units in Skeletal Muscle. J. Cell Biol..

[114] Protasi F., Takekura H., Wang Y., Chen S.R.W., Meissner G., Allen P.D., Franzini-Armstrong C. (2000). RYR1 and RYR3 Have Different Roles in the Assembly of Calcium Release Units of Skeletal Muscle. Biophys. J..

[115] Protasi F., Paolini C., Nakai J., Beam K.G., Franzini-Armstrong C., Allen P.D. (2002). Multiple Regions of RyR1 Mediate Functional and Structural Interactions with A1S-Dihydropyridine Receptors in Skeletal Muscle. Biophys. J..

[116] Nakai J., Ogura T., Protasi F., Franzini-Armstrong C., Allen P.D., Beam K.G. (1997). Functional Nonequality of the Cardiac and Skeletal Ryanodine Receptors. Proc. Natl. Acad. Sci. USA.

[117] Takekura H., Paolini C., Franzini-Armstrong C., Kugler G., Grabner M., Flucher B.E. (2004). Differential Contribution of Skeletal and Cardiac II-III Loop Sequences to the Assembly of Dihydropyridine-Receptor Arrays in Skeletal Muscle. Mol. Biol. Cell.

[118] MacLennan D.H., Wong P.T.S. (1971). Isolation of a Calcium-Sequestering Protein from Sarcoplasmic Reticulum. Proc. Natl. Acad. Sci. USA.

[119] MacLennan D.H., de Leon S. (1983). [45] Biosynthesis of Sarcoplasmic Reticulum Proteins. Methods Enzymol..

[120] Kim K.C., Caswell A.H., Talvenheimo J.A., Brandt N.R. (1990). Isolation of a Terminal Cisterna Protein Which May Link the Dihydropyridine Receptor to the Junctional Foot Protein in Skeletal Muscle. Biochemistry.

[121] Caswell A.H., Brandt N.R., Brunschwig J.P., Purkerson S. (1991). Localization and Partial Characterization of the Oligomeric Disulfide-Linked Molecular Weight 95,000 Protein (Triadin) Which Binds the Ryanodine and Dihydropyridine Receptors in Skeletal Muscle Triadic Vesicles. Biochemistry.

[122] Guo W., Campbell K.P. (1995). Association of Triadin with the Ryanodine Receptor and Calsequestrin in the Lumen of the Sarcoplasmic Reticulum. J. Biol. Chem..

[123] Zhang L., Kelley J., Schmeisser G., Kobayashi Y.M., Jones L.R. (1997). Complex Formation between Junctin, Triadin, Calsequestrin, and the Ryanodine Receptor. J. Biol. Chem..

[124] Takeshima H., Shimuta M., Komazaki S., Ohmi K., Nishi M., Iino M., Miyata A., Kangawa K. (1998). Mitsugumin29, a Novel Synaptophysin Family Member from the Triad Junction in Skeletal Muscle. Biochem. J..

[125] Zorzato F., Anderson A.A., Ohlendieck K., Froemming G., Guerrini R., Treves S. (2000). Identification of a Novel 45 KDa Protein (JP-45) from Rabbit Sarcoplasmic-Reticulum Junctional-Face Membrane. Biochem. J..

[126] Takeshima H. (2000). Junctophilins A Novel Family of Junctional Membrane Complex Proteins. Mol. Cell.

[127] Ríos E., Györke S. (2009). Calsequestrin, Triadin and More: The Molecules That Modulate Calcium Release in Cardiac and Skeletal Muscle. J. Physiol..

[128] Rebbeck R.T., Karunasekara Y., Board P.G., Beard N.A., Casarotto M.G., Dulhunty A.F. (2014). Skeletal Muscle Excitation–Contraction Coupling: Who Are the Dancing Partners?. Int. J. Biochem. Cell Biol..

[129] Franzini-armstrong C., Porter K.R. (1964). Sarcolemmal Invaginations Constituting the T System in Fish Muscle Fibers. J. Cell Biol..

[130] Franzini-Armstrong C. (1999). The Sarcoplasmic Reticulum and the Control of Muscle Contraction. FASEB J..

[131] Paolini C., Protasi F., Franzini-Armstrong C. (2004). The Relative Position of RyR Feet and DHPR Tetrads in Skeletal Muscle. J. Mol. Biol..

[132] Ikemoto N., Ronjat M., Meszaros L.G., Koshita M. (1989). Postulated Role of Calsequestrin in the Regulation of Calcium Release from Sarcoplasmic Reticulum. Biochemistry.

[133] Franzini-Armstrong C., Kenney L.J., Varriano-Marston E. (1987). The Structure of Calsequestrin in Triads of Vertebrate Skeletal Muscle: A Deep-Etch Study. J. Cell Biol..

[134] Boncompagni S., Protasi F., Franzini-Armstrong C. (2012). Sequential Stages in the Age-Dependent Gradual Formation and Accumulation of Tubular Aggregates in Fast Twitch Muscle Fibers: SERCA and Calsequestrin Involvement. Age.

[135] Hurne A.M., O’Brien J.J., Wingrove D., Cherednichenko G., Allen P.D., Beam K.G., Pessah I.N. (2005). Ryanodine Receptor Type 1 (RyR1) Mutations C4958S and C4961S Reveal Excitation-Coupled Calcium Entry (ECCE) Is Independent of Sarcoplasmic Reticulum Store Depletion. J. Biol. Chem..

[136] Bannister R.A., Pessah I.N., Beam K.G. (2009). The Skeletal L-Type Ca^2+^ Current Is a Major Contributor to Excitation-Coupled Ca^2+^ Entry. J. Gen. Physiol..

[137] Cherednichenko G., Ward C.W., Feng W., Cabrales E., Michaelson L., Samso M., López J.R., Allen P.D., Pessah I.N. (2008). Enhanced Excitation-Coupled Calcium Entry in Myotubes Expressing Malignant Hyperthermia Mutation R163C Is Attenuated by Dantrolene. Mol. Pharmacol..

[138] Hogan P.G., Rao A. (2015). Store-Operated Calcium Entry: Mechanisms and Modulation. Biochem. Biophys. Res. Commun..

[139] Hidalgo C., González M.E., García A.M. (1986). Calcium Transport in Transverse Tubules Isolated from Rabbit Skeletal Muscle. Biochim. Biophys. Acta (BBA)—Biomembr..

[140] Cheng A.J., Place N., Westerblad H. (2018). Molecular Basis for Exercise-Induced Fatigue: The Importance of Strictly Controlled Cellular Ca^2+^ Handling. Cold Spring Harb. Perspect. Med..

[141] Brini M., Carafoli E. (2011). The Plasma Membrane Ca^2+^ ATPase and the Plasma Membrane Sodium Calcium Exchanger Cooperate in the Regulation of Cell Calcium. Cold Spring Harb. Perspect. Biol..

[142] Balnave C.D., Allen D.G. (1998). Evidence for Na^+^/Ca^2+^ Exchange in Intact Single Skeletal Muscle Fibers from the Mouse. Am. J. Physiol.-Cell Physiol..

[143] Roos J., DiGregorio P.J., Yeromin A.V., Ohlsen K., Lioudyno M., Zhang S., Safrina O., Kozak J.A., Wagner S.L., Cahalan M.D. (2005). STIM1, an Essential and Conserved Component of Store-Operated Ca^2+^ Channel Function. J. Cell Biol..

[144] Liou J., Kim M.L., Do Heo W., Jones J.T., Myers J.W., Ferrell J.E., Meyer T. (2005). STIM Is a Ca^2+^ Sensor Essential for Ca^2+^-Store-Depletion-Triggered Ca^2+^ Influx. Curr. Biol..

[145] Feske S., Gwack Y., Prakriya M., Srikanth S., Puppel S.-H., Tanasa B., Hogan P.G., Lewis R.S., Daly M., Rao A. (2006). A Mutation in Orai1 Causes Immune Deficiency by Abrogating CRAC Channel Function. Nature.

[146] Vig M., Peinelt C., Beck A., Koomoa D.L., Rabah D., Koblan-Huberson M., Kraft S., Turner H., Fleig A., Penner R. (2006). CRACM1 Is a Plasma Membrane Protein Essential for Store-Operated Ca^2+^ Entry. Science.

[147] Lewis R.S. (2007). The Molecular Choreography of a Store-Operated Calcium Channel. Nature.

[148] Zhang L., Wang L., Li S., Xue J., Luo D. (2016). Calsequestrin-1 Regulates Store-Operated Ca^2+^ Entry by Inhibiting STIM1 Aggregation. Cell. Physiol. Biochem..

[149] Wang L., Zhang L., Li S., Zheng Y., Yan X., Chen M., Wang H., Putney J.W., Luo D. (2015). Retrograde Regulation of STIM1-Orai1 Interaction and Store-Operated Ca^2+^ Entry by Calsequestrin. Sci. Rep..

[150] Shin D.W., Pan Z., Kim E.K., Lee J.M., Bhat M.B., Parness J., Kim D.H., Ma J. (2003). A Retrograde Signal from Calsequestrin for the Regulation of Store-Operated Ca^2+^ Entry in Skeletal Muscle. J. Biol. Chem..

[151] Liou J., Fivaz M., Inoue T., Meyer T. (2007). Live-Cell Imaging Reveals Sequential Oligomerization and Local Plasma Membrane Targeting of Stromal Interaction Molecule 1 after Ca^2+^ Store Depletion. Proc. Natl. Acad. Sci. USA.

[152] Wu M.M., Buchanan J., Luik R.M., Lewis R.S. (2006). Ca^2+^ Store Depletion Causes STIM1 to Accumulate in ER Regions Closely Associated with the Plasma Membrane. J. Cell Biol..

[153] Luik R.M., Wu M.M., Buchanan J., Lewis R.S. (2006). The Elementary Unit of Store-Operated Ca^2+^ Entry: Local Activation of CRAC Channels by STIM1 at ER–Plasma Membrane Junctions. J. Cell Biol..

[154] Zhang S.L., Yeromin A.V., Zhang X.H.-F., Yu Y., Safrina O., Penna A., Roos J., Stauderman K.A., Cahalan M.D. (2006). Genome-Wide RNAi Screen of Ca^2+^ Influx Identifies Genes That Regulate Ca^2+^ Release-Activated Ca^2+^ Channel Activity. Proc. Natl. Acad. Sci. USA.

[155] Wu M.M., Covington E.D., Lewis R.S. (2014). Single-Molecule Analysis of Diffusion and Trapping of STIM1 and Orai1 at Endoplasmic Reticulum–Plasma Membrane Junctions. Mol. Biol. Cell.

[156] Luik R.M., Wang B., Prakriya M., Wu M.M., Lewis R.S. (2008). Oligomerization of STIM1 Couples ER Calcium Depletion to CRAC Channel Activation. Nature.

[157] Stiber J., Hawkins A., Zhang Z.-S., Wang S., Burch J., Graham V., Ward C.C., Seth M., Finch E., Malouf N. (2008). STIM1 Signalling Controls Store-Operated Calcium Entry Required for Development and Contractile Function in Skeletal Muscle. Nat. Cell Biol..

[158] Lyfenko A.D., Dirksen R.T. (2008). Differential Dependence of Store-operated and Excitation-coupled Ca^2+^ Entry in Skeletal Muscle on STIM1 and Orai1. J. Physiol..

[159] Carrell E.M., Coppola A.R., McBride H.J., Dirksen R.T. (2016). Orai1 Enhances Muscle Endurance by Promoting Fatigue-resistant Type I Fiber Content but Not through Acute Store-operated Ca^2+^ Entry. FASEB J..

[160] Avila-Medina J., Mayoral-Gonzalez I., Dominguez-Rodriguez A., Gallardo-Castillo I., Ribas J., Ordoñez A., Rosado J.A., Smani T. (2018). The Complex Role of Store Operated Calcium Entry Pathways and Related Proteins in the Function of Cardiac, Skeletal and Vascular Smooth Muscle Cells. Front. Physiol..

[161] Seth M., Li T., Graham V., Burch J., Finch E., Stiber J.A., Rosenberg P.B. (2012). Dynamic Regulation of Sarcoplasmic Reticulum Ca(^2+^) Stores by Stromal Interaction Molecule 1 and Sarcolipin during Muscle Differentiation. Dev. Dyn..

[162] Kiviluoto S., Decuypere J.-P., De Smedt H., Missiaen L., Parys J.B., Bultynck G. (2011). STIM1 as a Key Regulator for Ca^2+^ Homeostasis in Skeletal-Muscle Development and Function. Skelet. Muscle.

[163] Launikonis B.S., Stephenson D.G., Friedrich O. (2009). Rapid Ca^2+^ Flux through the Transverse Tubular Membrane, Activated by Individual Action Potentials in Mammalian Skeletal Muscle. J. Physiol..

[164] Edwards J.N., Murphy R.M., Cully T.R., von Wegner F., Friedrich O., Launikonis B.S. (2010). Ultra-Rapid Activation and Deactivation of Store-Operated Ca^2+^ Entry in Skeletal Muscle. Cell Calcium.

[165] Darbellay B., Arnaudeau S., König S., Jousset H., Bader C., Demaurex N., Bernheim L. (2009). STIM1- and Orai1-Dependent Store-Operated Calcium Entry Regulates Human Myoblast Differentiation. J. Biol. Chem..

[166] Darbellay B., Arnaudeau S., Bader C.R., Konig S., Bernheim L. (2011). STIM1L Is a New Actin-Binding Splice Variant Involved in Fast Repetitive Ca^2+^ Release. J. Cell Biol..

[167] Bootman M.D., Collins T.J., Mackenzie L., Roderick H.L., Berridge M.J., Peppiatt C.M. (2002). 2-Aminoethoxydiphenyl Borate (2-APB) Is a Reliable Blocker of Store-Operated Ca^2+^ Entry but an Inconsistent Inhibitor of InsP_3_-Induced Ca^2+^ Release. FASEB J..

[168] Zitt C., Strauss B., Schwarz E.C., Spaeth N., Rast G., Hatzelmann A., Hoth M. (2004). Potent Inhibition of Ca^2+^ Release-Activated Ca^2+^ Channels and T-Lymphocyte Activation by the Pyrazole Derivative BTP2. J. Biol. Chem..

[169] Girolami B., Serano M., Di Fonso A., Paolini C., Pietrangelo L., Protasi F. (2023). Searching for Mechanisms Underlying the Assembly of Calcium Entry Units: The Role of Temperature and PH. Int. J. Mol. Sci..

[170] Soboloff J., Rothberg B.S., Madesh M., Gill D.L. (2012). STIM Proteins: Dynamic Calcium Signal Transducers. Nat. Rev. Mol. Cell Biol..

[171] Mancarella S., Wang Y., Deng X., Landesberg G., Scalia R., Panettieri R.A., Mallilankaraman K., Tang X.D., Madesh M., Gill D.L. (2011). Hypoxia-Induced Acidosis Uncouples the STIM-Orai Calcium Signaling Complex. J. Biol. Chem..

[172] Protasi F., Pietrangelo L., Boncompagni S. (2021). Calcium Entry Units (CEUs): Perspectives in Skeletal Muscle Function and Disease. J. Muscle Res. Cell Motil..

[173] Melzer W. (2020). ECC Meets CEU—New Focus on the Backdoor for Calcium Ions in Skeletal Muscle Cells. J. Gen. Physiol..

[174] Paolini C., Quarta M., Nori A., Boncompagni S., Canato M., Volpe P., Allen P.D., Reggiani C., Protasi F. (2007). Reorganized Stores and Impaired Calcium Handling in Skeletal Muscle of Mice Lacking Calsequestrin-1. J. Physiol..

[175] Protasi F., Paolini C., Dainese M. (2009). Calsequestrin-1: A New Candidate Gene for Malignant Hyperthermia and Exertional/Environmental Heat Stroke. J. Physiol..

[176] Dainese M., Quarta M., Lyfenko A.D., Paolini C., Canato M., Reggiani C., Dirksen R.T., Protasi F. (2009). Anesthetic-and Heat-induced Sudden Death in Calsequestrin-1-knockout Mice. FASEB J..

[177] Canato M., Scorzeto M., Giacomello M., Protasi F., Reggiani C., Stienen G.J.M. (2010). Massive Alterations of Sarcoplasmic Reticulum Free Calcium in Skeletal Muscle Fibers Lacking Calsequestrin Revealed by a Genetically Encoded Probe. Proc. Natl. Acad. Sci. USA.

[178] Murzilli S., Serano M., Pietrangelo L., Protasi F., Paolini C. (2023). Structural Adaptation of the Excitation–Contraction Coupling Apparatus in Calsequestrin1-Null Mice during Postnatal Development. Biology.

[179] Brotto M. (2011). Aging, Sarcopenia and Store-Operated Calcium Entry. Cell Cycle.

[180] Thornton A.M., Zhao X., Weisleder N., Brotto L.S., Bougoin S., Nosek T.M., Reid M., Hardin B., Pan Z., Ma J. (2011). Store-Operated Ca^2+^ Entry (SOCE) Contributes to Normal Skeletal Muscle Contractility in Young but Not in Aged Skeletal Muscle. Aging.

[181] Boncompagni S., Pecorai C., Michelucci A., Pietrangelo L., Protasi F. (2021). Long-Term Exercise Reduces Formation of Tubular Aggregates and Promotes Maintenance of Ca^2+^ Entry Units in Aged Muscle. Front. Physiol..

[182] Michelucci A., Paolini C., Canato M., Wei-Lapierre L., Pietrangelo L., De Marco A., Reggiani C., Dirksen R.T., Protasi F. (2015). Antioxidants Protect Calsequestrin-1 Knockout Mice from Halothane- and Heat-Induced Sudden Death. Anesthesiology.

[183] Hoogendijk E.O., Afilalo J., Ensrud K.E., Kowal P., Onder G., Fried L.P. (2019). Frailty: Implications for Clinical Practice and Public Health. Lancet.

[184] Cruz-Jentoft A.J., Sayer A.A. (2019). Sarcopenia. Lancet.

[185] Blane D., Netuveli G., Montgomery S.M. (2008). Quality of Life, Health and Physiological Status and Change at Older Ages. Soc. Sci. Med..

[186] Young A. (1997). Ageing and Physiological Functions. Philos. Trans. R. Soc. Lond. B Biol. Sci..

[187] Vandervoort A.A. (2002). Aging of the Human Neuromuscular System. Muscle Nerve.

[188] Grimby G., Saltin B. (1983). The Ageing Muscle. Clin. Physiol..

[189] Clark B.C. (2019). Neuromuscular Changes with Aging and Sarcopenia. J. Frailty Aging.

[190] Schneider E.L., Guralnik J.M. (1990). The Aging of America. Impact on Health Care Costs. JAMA.

[191] Janssen I., Shepard D.S., Katzmarzyk P.T., Roubenoff R. (2004). The Healthcare Costs of Sarcopenia in the United States. J. Am. Geriatr. Soc..

[192] Goates S., Du K., Arensberg M.B., Gaillard T., Guralnik J., Pereira S.L. (2019). Economic Impact of Hospitalizations in US Adults with Sarcopenia. J. Frailty Aging.

[193] Abellan van Kan G. (2009). Epidemiology and Consequences of Sarcopenia. J. Nutr. Health Aging.

[194] Evans W.J. (1995). What Is Sarcopenia?. J. Gerontol. Ser. A Biol. Sci. Med. Sci..

[195] Wiedmer P., Jung T., Castro J.P., Pomatto L.C.D., Sun P.Y., Davies K.J.A., Grune T. (2021). Sarcopenia—Molecular Mechanisms and Open Questions. Ageing Res. Rev..

[196] Rosenberg I.H. (1989). Summary Comments. Am. J. Clin. Nutr..

[197] Roubenoff R., Hughes V.A. (2000). Sarcopenia: Current Concepts. J. Gerontol. A Biol. Sci. Med. Sci..

[198] Alchin D.R. (2014). Sarcopenia: Describing Rather than Defining a Condition. J. Cachexia Sarcopenia Muscle.

[199] Mitchell W.K., Williams J., Atherton P., Larvin M., Lund J., Narici M. (2012). Sarcopenia, Dynapenia, and the Impact of Advancing Age on Human Skeletal Muscle Size and Strength; a Quantitative Review. Front. Physiol..

[200] Luff A.R. (1998). Age-associated Changes in the Innervation of Muscle Fibers and Changes in the Mechanical Properties of Motor Units. Ann. N. Y. Acad. Sci..

[201] Conley K.E., Jubrias S.A., Esselman P.C. (2000). Oxidative Capacity and Ageing in Human Muscle. J. Physiol..

[202] Damanti S., Senini E., De Lorenzo R., Merolla A., Santoro S., Festorazzi C., Messina M., Vitali G., Sciorati C., Manfredi A.A. (2025). Molecular Constraints of Sarcopenia in the Ageing Muscle. Front. Aging.

[203] Rygiel K.A., Picard M., Turnbull D.M. (2016). The Ageing Neuromuscular System and Sarcopenia: A Mitochondrial Perspective. J. Physiol..

[204] Marzetti E., Calvani R., Coelho-Júnior H.J., Landi F., Picca A. (2024). Mitochondrial Quantity and Quality in Age-Related Sarcopenia. Int. J. Mol. Sci..

[205] Wilkinson D.J., Piasecki M., Atherton P.J. (2018). The Age-Related Loss of Skeletal Muscle Mass and Function: Measurement and Physiology of Muscle Fibre Atrophy and Muscle Fibre Loss in Humans. Ageing Res. Rev..

[206] Tieland M., Trouwborst I., Clark B.C. (2018). Skeletal Muscle Performance and Ageing. J. Cachexia Sarcopenia Muscle.

[207] Delbono O., O’Rourke K.S., Ettinger W.H. (1995). Excitation-Calcium Release Uncoupling in Aged Single Human Skeletal Muscle Fibers. J. Membr. Biol..

[208] Delbono O. (2011). Expression and Regulation of Excitation-Contraction Coupling Proteins in Aging Skeletal Muscle. Curr. Aging Sci..

[209] Renganathan M., Messi M.L., Delbono O. (1997). Dihydropyridine Receptor-Ryanodine Receptor Uncoupling in Aged Skeletal Muscle. J. Membr. Biol..

[210] Ryan M., Ohlendieck K. (2004). Excitation-Contraction Uncoupling and Sarcopenia. Basic. Appl. Myol..

[211] Ryan M., Butler-Browne G., Erzen I., Mouly V., Thornell L.-E., Wernig A., Ohlendieck K. (2003). Persistent Expression of the Alpha1S-Dihydropyridine Receptor in Aged Human Skeletal Muscle: Implications for the Excitation-Contraction Uncoupling Hypothesis of Sarcopenia. Int. J. Mol. Med..

[212] Zampieri S., Pietrangelo L., Loefler S., Fruhmann H., Vogelauer M., Burggraf S., Pond A., Grim-Stieger M., Cvecka J., Sedliak M. (2015). Lifelong Physical Exercise Delays Age-Associated Skeletal Muscle Decline. J. Gerontol. A Biol. Sci. Med. Sci..

[213] Boncompagni S., d’Amelio L., Fulle S., Fano G., Protasi F. (2006). Progressive Disorganization of the Excitation-Contraction Coupling Apparatus in Aging Human Skeletal Muscle as Revealed by Electron Microscopy: A Possible Role in the Decline of Muscle Performance. J. Gerontol. A Biol. Sci. Med. Sci..

[214] Vue Z., Garza-Lopez E., Neikirk K., Katti P., Vang L., Beasley H., Shao J., Marshall A.G., Crabtree A., Murphy A.C. (2023). 3D Reconstruction of Murine Mitochondria Reveals Changes in Structure during Aging Linked to the MICOS Complex. Aging Cell.

[215] Scudese E., Marshall A.G., Vue Z., Exil V., Rodriguez B.I., Demirci M., Vang L., López E.G., Neikirk K., Shao B. (2025). 3D Mitochondrial Structure in Aging Human Skeletal Muscle: Insights Into MFN-2-Mediated Changes. Aging Cell.

[216] Vincent A.E., White K., Davey T., Philips J., Ogden R.T., Lawless C., Warren C., Hall M.G., Ng Y.S., Falkous G. (2019). Quantitative 3D Mapping of the Human Skeletal Muscle Mitochondrial Network. Cell Rep..

[217] Cheng H., Lederer W.J., Cannell M.B. (1993). Calcium Sparks: Elementary Events Underlying Excitation-Contraction Coupling in Heart Muscle. Science.

[218] Cheng H., Lederer W.J. (2008). Calcium Sparks. Physiol. Rev..

[219] Tsugorka A., Ríos E., Blatter L.A. (1995). Imaging Elementary Events of Calcium Release in Skeletal Muscle Cells. Science.

[220] Ríos E. (2002). Ca^2+^ Release Flux Underlying Ca^2+^ Transients and Ca^2+^ Sparks in Skeletal Muscle. Front. Biosci..

[221] Hollingworth S., Zhao M., Baylor S.M. (1996). The Amplitude and Time Course of the Myoplasmic Free [Ca^2+^] Transient in Fast-Twitch Fibers of Mouse Muscle. J. Gen. Physiol..

[222] Csernoch L., Zhou J., Stern M.D., Brum G., Ríos E. (2004). The Elementary Events of Ca^2+^ Release Elicited by Membrane Depolarization in Mammalian Muscle. J. Physiol..

[223] Apostol S., Ursu D., Lehmann-Horn F., Melzer W. (2009). Local Calcium Signals Induced by Hyper-Osmotic Stress in Mammalian Skeletal Muscle Cells. J. Muscle Res. Cell Motil..

[224] Salviati G., Pierobon-Bormioli S., Betto R., Damiani E., Angelini C., Ringel S.P., Salvatori S., Margreth A. (1985). Tubular Aggregates: Sarcoplasmic Reticulum Origin, Calcium Storage Ability, and Functional Implications. Muscle Nerve.

[225] Rosenberg N.L. (1985). Tubular Aggregates. Arch. Neurol..

[226] Pierobon-Bormioli S., Armani M., Ringel S.P., Angelini C., Vergani L., Betto R., Salviati G. (1985). Familial Neuromuscular Disease with Tubular Aggregates. Muscle Nerve.

[227] Morgan-Hughes J.A. (1998). Tubular Aggregates in Skeletal Muscle: Their Functional Significance and Mechanisms of Pathogenesis. Curr. Opin. Neurol..

[228] Engel W.K., Bishop D.W., Cunningham G.G. (1970). Tubular Aggregates in Type II Muscle Fibers: Ultrastructural and Histochemical Correlation. J. Ultrastruct. Res..

[229] Groot J.G., Arts W.F. (1982). Familial Myopathy with Tubular Aggregates. J. Neurol..

[230] Walter M.C., Rossius M., Zitzelsberger M., Vorgerd M., Müller-Felber W., Ertl-Wagner B., Zhang Y., Brinkmeier H., Senderek J., Schoser B. (2015). 50 Years to Diagnosis: Autosomal Dominant Tubular Aggregate Myopathy Caused by a Novel STIM1 Mutation. Neuromuscul. Disord..

[231] Okuma H., Saito F., Mitsui J., Hara Y., Hatanaka Y., Ikeda M., Shimizu T., Matsumura K., Shimizu J., Tsuji S. (2016). Tubular Aggregate Myopathy Caused by a Novel Mutation in the Cytoplasmic Domain of *STIM1*. Neurol. Genet..

[232] Nesin V., Wiley G., Kousi M., Ong E.-C., Lehmann T., Nicholl D.J., Suri M., Shahrizaila N., Katsanis N., Gaffney P.M. (2014). Activating Mutations in *STIM1* and *ORAI1* Cause Overlapping Syndromes of Tubular Myopathy and Congenital Miosis. Proc. Natl. Acad. Sci. USA.

[233] Endo Y., Noguchi S., Hara Y., Hayashi Y.K., Motomura K., Miyatake S., Murakami N., Tanaka S., Yamashita S., Kizu R. (2015). Dominant Mutations in ORAI1 Cause Tubular Aggregate Myopathy with Hypocalcemia via Constitutive Activation of Store-Operated Ca^2+^ Channels. Hum. Mol. Genet..

[234] Böhm J., Chevessier F., De Paula A.M., Koch C., Attarian S., Feger C., Hantaï D., Laforêt P., Ghorab K., Vallat J.-M. (2013). Constitutive Activation of the Calcium Sensor STIM1 Causes Tubular-Aggregate Myopathy. Am. J. Human. Genet..

[235] Böhm J., Bulla M., Urquhart J.E., Malfatti E., Williams S.G., O’Sullivan J., Szlauer A., Koch C., Baranello G., Mora M. (2017). ORAI1 Mutations with Distinct Channel Gating Defects in Tubular Aggregate Myopathy. Hum. Mutat..

[236] Barone V., Del Re V., Gamberucci A., Polverino V., Galli L., Rossi D., Costanzi E., Toniolo L., Berti G., Malandrini A. (2017). Identification and Characterization of Three Novel Mutations in the *CASQ1* Gene in Four Patients with Tubular Aggregate Myopathy. Hum. Mutat..

[237] Chevessier F., Marty I., Paturneau-Jouas M., Hantai D., Verdière-Sahuqué M. (2004). Tubular Aggregates Are from Whole Sarcoplasmic Reticulum Origin: Alterations in Calcium Binding Protein Expression in Mouse Skeletal Muscle during Aging. Neuromuscul. Disord..

[238] Schiaffino S., Severin E., Cantini M., Sartore S. (1977). Tubular Aggregates Induced by Anoxia in Isolated Rat Skeletal Muscle. Lab. Investig..

[239] Zhao X., Weisleder N., Thornton A., Oppong Y., Campbell R., Ma J., Brotto M. (2008). Compromised Store-operated Ca^2+^ Entry in Aged Skeletal Muscle. Aging Cell.

[240] Guo X., Zhou Y., Li X., Mu J. (2025). Resistance Exercise Training Improves Disuse-Induced Skeletal Muscle Atrophy in Humans: A Meta-Analysis of Randomized Controlled Trials. BMC Musculoskelet. Disord..

[241] Stec M.J., Thalacker-Mercer A., Mayhew D.L., Kelly N.A., Tuggle S.C., Merritt E.K., Brown C.J., Windham S.T., Dell’Italia L.J., Bickel C.S. (2017). Randomized, Four-Arm, Dose-Response Clinical Trial to Optimize Resistance Exercise Training for Older Adults with Age-Related Muscle Atrophy. Exp. Gerontol..

[242] Furrer R., Handschin C. (2024). Molecular Aspects of the Exercise Response and Training Adaptation in Skeletal Muscle. Free Radic. Biol. Med..

[243] Brightwell C.R., Markofski M.M., Moro T., Fry C.S., Porter C., Volpi E., Rasmussen B.B. (2019). Moderate-Intensity Aerobic Exercise Improves Skeletal Muscle Quality in Older Adults. Transl. Sports Med..

[244] Harber M.P., Konopka A.R., Douglass M.D., Minchev K., Kaminsky L.A., Trappe T.A., Trappe S. (2009). Aerobic Exercise Training Improves Whole Muscle and Single Myofiber Size and Function in Older Women. Am. J. Physiol. Regul. Integr. Comp. Physiol..

[245] Bodine S.C., Stitt T.N., Gonzalez M., Kline W.O., Stover G.L., Bauerlein R., Zlotchenko E., Scrimgeour A., Lawrence J.C., Glass D.J. (2001). Akt/MTOR Pathway Is a Crucial Regulator of Skeletal Muscle Hypertrophy and Can Prevent Muscle Atrophy in Vivo. Nat. Cell Biol..

[246] Jäger S., Handschin C., St.-Pierre J., Spiegelman B.M. (2007). AMP-Activated Protein Kinase (AMPK) Action in Skeletal Muscle via Direct Phosphorylation of PGC-1α. Proc. Natl. Acad. Sci. USA.

[247] Hawley J.A. (2009). Molecular Responses to Strength and Endurance Training: Are They Incompatible?. Appl. Physiol. Nutr. Metab..

[248] Camera D.M., Smiles W.J., Hawley J.A. (2016). Exercise-Induced Skeletal Muscle Signaling Pathways and Human Athletic Performance. Free Radic. Biol. Med..

[249] Polito M.D., Papst R.R., Farinatti P. (2021). Moderators of Strength Gains and Hypertrophy in Resistance Training: A Systematic Review and Meta-Analysis. J. Sports Sci..

[250] Moro T., Brightwell C.R., Volpi E., Rasmussen B.B., Fry C.S. (2020). Resistance Exercise Training Promotes Fiber Type-Specific Myonuclear Adaptations in Older Adults. J. Appl. Physiol..

[251] Straight C.R., Fedewa M.V., Toth M.J., Miller M.S. (2020). Improvements in Skeletal Muscle Fiber Size with Resistance Training Are Age-Dependent in Older Adults: A Systematic Review and Meta-Analysis. J. Appl. Physiol..

[252] Li J., Zhang S., Li C., Zhang X., Shan Y., Zhang Z., Bo H., Zhang Y. (2024). Endurance Exercise-Induced Histone Methylation Modification Involved in Skeletal Muscle Fiber Type Transition and Mitochondrial Biogenesis. Sci. Rep..

[253] Verdijk L.B., Gleeson B.G., Jonkers R.A.M., Meijer K., Savelberg H.H.C.M., Dendale P., van Loon L.J.C. (2009). Skeletal Muscle Hypertrophy Following Resistance Training Is Accompanied by a Fiber Type-Specific Increase in Satellite Cell Content in Elderly Men. J. Gerontol. A Biol. Sci. Med. Sci..

[254] Zampieri S., Mammucari C., Romanello V., Barberi L., Pietrangelo L., Fusella A., Mosole S., Gherardi G., Höfer C., Löfler S. (2016). Physical Exercise in Aging Human Skeletal Muscle Increases Mitochondrial Calcium Uniporter Expression Levels and Affects Mitochondria Dynamics. Physiol. Rep..

[255] Mosole S., Carraro U., Kern H., Loefler S., Fruhmann H., Vogelauer M., Burggraf S., Mayr W., Krenn M., Paternostro-Sluga T. (2014). Long-Term High-Level Exercise Promotes Muscle Reinnervation With Age. J. Neuropathol. Exp. Neurol..

[256] Tintignac L.A., Brenner H.-R., Rüegg M.A. (2015). Mechanisms Regulating Neuromuscular Junction Development and Function and Causes of Muscle Wasting. Physiol. Rev..

[257] Jang Y.C., Van Remmen H. (2011). Age-Associated Alterations of the Neuromuscular Junction. Exp. Gerontol..

[258] Schiaffino S., Dyar K.A., Ciciliot S., Blaauw B., Sandri M. (2013). Mechanisms Regulating Skeletal Muscle Growth and Atrophy. FEBS J..

[259] Iyer S.R., Shah S.B., Lovering R.M. (2021). The Neuromuscular Junction: Roles in Aging and Neuromuscular Disease. Int. J. Mol. Sci..

[260] Roos M.R., Rice C.L., Vandervoort A.A. (1997). Age-Related Changes in Motor Unit Function. Muscle Nerve.

[261] Doherty T.J., Vandervoort A.A., Taylor A.W., Brown W.F. (1993). Effects of Motor Unit Losses on Strength in Older Men and Women. J. Appl. Physiol..

[262] Larsson L. (1998). The Age-related Motor Disability: Underlying Mechanisms in Skeletal Muscle at the Motor Unit, Cellular and Molecular Level. Acta Physiol. Scand..

[263] Aagaard P., Suetta C., Caserotti P., Magnusson S.P., Kjær M. (2010). Role of the Nervous System in Sarcopenia and Muscle Atrophy with Aging: Strength Training as a Countermeasure. Scand. J. Med. Sci. Sports.

[264] Lexell J. (1997). Evidence for Nervous System Degeneration with Advancing Age. J. Nutr..

[265] Chai R.J., Vukovic J., Dunlop S., Grounds M.D., Shavlakadze T. (2011). Striking Denervation of Neuromuscular Junctions without Lumbar Motoneuron Loss in Geriatric Mouse Muscle. PLoS ONE.

[266] Pellegrino C., Franzini C. (1963). An Electron Microscope Study of Denervation Atrophy in Red and White Skeletal Muscle Fibers. J. Cell Biol..

[267] Engel A.G., Franzini-Armstrong C. (2004). Myology.

[268] Carlson B.M. (2014). The Biology of Long-Term Denervated Skeletal Muscle. Eur. J. Transl. Myol..

[269] Carraro U., Kern H., Gava P., Hofer C., Loefler S., Gargiulo P., Mosole S., Zampieri S., Gobbo V., Ravara B. (2015). Biology of Muscle Atrophy and of Its Recovery by FES in Aging and Mobility Impairments: Roots and by-Products. Eur. J. Transl. Myol..

[270] Sirago G., Pellegrino M.A., Bottinelli R., Franchi M.V., Narici M. (2023). V Loss of Neuromuscular Junction Integrity and Muscle Atrophy in Skeletal Muscle Disuse. Ageing Res. Rev..

[271] Takekura H., Kasuga N., Kitada K., Yoshioka T. (1996). Morphological Changes in the Triads and Sarcoplasmic Reticulum of Rat Slow and Fast Muscle Fibres Following Denervation and Immobilization. J. Muscle Res. Cell Motil..

[272] Mödlin M., Forstner C., Hofer C., Mayr W., Richter W., Carraro U., Protasi F., Kern H. (2005). Electrical Stimulation of Denervated Muscles: First Results of a Clinical Study. Artif. Organs.

[273] Ashley Z., Salmons S., Boncompagni S., Protasi F., Russold M., Lanmuller H., Mayr W., Sutherland H., Jarvis J.C. (2007). Effects of Chronic Electrical Stimulation on Long-Term Denervated Muscles of the Rabbit Hind Limb. J. Muscle Res. Cell Motil..

[274] Ashley Z., Sutherland H., Lanmüller H., Russold M.F., Unger E., Bijak M., Mayr W., Boncompagni S., Protasi F., Salmons S. (2007). Atrophy, but Not Necrosis, in Rabbit Skeletal Muscle Denervated for Periods up to One Year. Am. J. Physiol.-Cell Physiol..

[275] Squecco R., Carraro U., Kern H., Pond A., Adami N., Biral D., Vindigni V., Boncompagni S., Pietrangelo T., Bosco G. (2009). A Subpopulation of Rat Muscle Fibers Maintains an Assessable Excitation-Contraction Coupling Mechanism After Long-Standing Denervation Despite Lost Contractility. J. Neuropathol. Exp. Neurol..

[276] Kern H., Boncompagni S., Rossini K., Mayr W., Fanò G., Zanin M.E., Podhorska-Okolow M., Protasi F., Carraro U. (2004). Long-Term Denervation in Humans Causes Degeneration of Both Contractile and Excitation-Contraction Coupling Apparatus, Which Is Reversible by Functional Electrical Stimulation (FES): A Role for Myofiber Regeneration?. J. Neuropathol. Exp. Neurol..

[277] Boncompagni S., Kern H., Rossini K., Hofer C., Mayr W., Carraro U., Protasi F. (2007). Structural Differentiation of Skeletal Muscle Fibers in the Absence of Innervation in Humans. Proc. Natl. Acad. Sci. USA.

[278] Kern H., Carraro U., Adami N., Hofer C., Loefler S., Vogelauer M., Mayr W., Rupp R., Zampieri S. (2010). One Year of Home-Based Daily FES in Complete Lower Motor Neuron Paraplegia: Recovery of Tetanic Contractility Drives the Structural Improvements of Denervated Muscle. Neurol. Res..

[279] Flucher B.E., Takekura H., Franzini-Armstrong C. (1993). Development of the Excitation-Contraction Coupling Apparatus in Skeletal Muscle: Association of Sarcoplasmic Reticulum and Transverse Tubules with Myofibrils. Dev. Biol..

[280] Pietrangelo L., Brasile A., Girolami B., Ravara B., Protasi F. (2023). Mimicking disuse and rehabilitation in a mouse model. Eur. J. Transl. Myol..

[281] Doucet B.M., Lam A., Griffin L. (2012). Neuromuscular Electrical Stimulation for Skeletal Muscle Function. Yale J. Biol. Med..

[282] Atkins K.D., Bickel C.S. (2021). Effects of Functional Electrical Stimulation on Muscle Health after Spinal Cord Injury. Curr. Opin. Pharmacol..

[283] Karamian B.A., Siegel N., Nourie B., Serruya M.D., Heary R.F., Harrop J.S., Vaccaro A.R. (2022). The Role of Electrical Stimulation for Rehabilitation and Regeneration after Spinal Cord Injury. J. Orthop. Traumatol..

[284] Kern H., Barberi L., Löfler S., Sbardella S., Burggraf S., Fruhmann H., Carraro U., Mosole S., Sarabon N., Vogelauer M. (2014). Electrical Stimulation Counteracts Muscle Decline in Seniors. Front. Aging Neurosci..

[285] Iodice P., Boncompagni S., Pietrangelo L., Galli L., Pierantozzi E., Rossi D., Fusella A., Caulo M., Kern H., Sorrentino V. (2019). Functional Electrical Stimulation: A Possible Strategy to Improve Muscle Function in Central Core Disease?. Front. Neurol..

[286] Carraro U., Boncompagni S., Gobbo V., Rossini K., Zampieri S., Mosole S., Ravara B., Nori A., Stramare R., Ambrosio F. (2015). Persistent Muscle Fiber Regeneration in Long Term Denervation. Past, Present, Future. Eur. J. Transl. Myol..

[287] Biral D., Kern H., Adami N., Boncompagni S., Protasi F., Carraro U. (2008). Atrophy-resistant fibers in permanent peripheral denervation of human skeletal muscle. Neurol. Res..

[288] Mo Y., Zhou Y., Chan H., Evans C., Maddocks M. (2023). The Association between Sedentary Behaviour and Sarcopenia in Older Adults: A Systematic Review and Meta-Analysis. BMC Geriatr..

[289] Hämäläinen O., Tirkkonen A., Savikangas T., Alén M., Sipilä S., Hautala A. (2024). Low Physical Activity Is a Risk Factor for Sarcopenia: A Cross-Sectional Analysis of Two Exercise Trials on Community-Dwelling Older Adults. BMC Geriatr..

[290] Luo J., Lee R.Y.W. (2025). Physical Activity Reduces the Incidence of Sarcopenia in Middle-Aged Adults. Ageing Int..

[291] Chen W., Wang L., You W., Shan T. (2021). Myokines Mediate the Cross Talk between Skeletal Muscle and Other Organs. J. Cell Physiol..

[292] Das D.K., Graham Z.A., Cardozo C.P. (2020). Myokines in Skeletal Muscle Physiology and Metabolism: Recent Advances and Future Perspectives. Acta Physiol..

[293] Severinsen M.C.K., Pedersen B.K. (2020). Muscle-Organ Crosstalk: The Emerging Roles of Myokines. Endocr. Rev..

[294] Grevendonk L., Connell N.J., McCrum C., Fealy C.E., Bilet L., Bruls Y.M.H., Mevenkamp J., Schrauwen-Hinderling V.B., Jörgensen J.A., Moonen-Kornips E. (2021). Impact of Aging and Exercise on Skeletal Muscle Mitochondrial Capacity, Energy Metabolism, and Physical Function. Nat. Commun..

[295] Short K.R., Bigelow M.L., Kahl J., Singh R., Coenen-Schimke J., Raghavakaimal S., Nair K.S. (2005). Decline in Skeletal Muscle Mitochondrial Function with Aging in Humans. Proc. Natl. Acad. Sci. USA.

[296] Broskey N.T., Greggio C., Boss A., Boutant M., Dwyer A., Schlueter L., Hans D., Gremion G., Kreis R., Boesch C. (2014). Skeletal Muscle Mitochondria in the Elderly: Effects of Physical Fitness and Exercise Training. J. Clin. Endocrinol. Metab..

